# Plant Natural Flavonoids Against Multidrug Resistant Pathogens

**DOI:** 10.1002/advs.202100749

**Published:** 2021-05-26

**Authors:** Meirong Song, Ying Liu, Tingting Li, Xiaojia Liu, Zhihui Hao, Shuangyang Ding, Pharkphoom Panichayupakaranant, Kui Zhu, Jianzhong Shen

**Affiliations:** ^1^ National Center for Veterinary Drug Safety Evaluation College of Veterinary Medicine China Agricultural University Beijing 100193 China; ^2^ Center of Research and Innovation of Chinese Traditional Veterinary Medicine China Agricultural University Beijing 100193 China; ^3^ Beijing Key Laboratory of Detection Technology for Animal‐Derived Food Safety and Beijing Laboratory for Food Quality and Safety China Agricultural University Beijing 100193 China; ^4^ Department of Pharmacognosy and Pharmaceutical Botany Faculty of Pharmaceutical Sciences Prince of Songkla University Hat‐Yai 90112 Thailand

**Keywords:** bacterial membrane, drug discovery, flavonoids, isopentenyl, multidrug‐resistant bacteria

## Abstract

The increasing emergence and dissemination of multidrug resistant (MDR) bacterial pathogens accelerate the desires for new antibiotics. Natural products dominate the preferred chemical scaffolds for the discovery of antibacterial agents. Here, the potential of natural flavonoids from plants against MDR bacteria, is demonstrated. Structure–activity relationship analysis shows the prenylation modulates the activity of flavonoids and obtains two compounds, *α*‐mangostin (AMG) and isobavachalcone (IBC). AMG and IBC not only display rapid bactericidal activity against Gram‐positive bacteria, but also restore the susceptibility of colistin against Gram‐negative pathogens. Mechanistic studies generally show such compounds bind to the phospholipids of bacterial membrane, and result in the dissipation of proton motive force and metabolic perturbations, through distinctive modes of action. The efficacy of AMG and IBC in four models associated with infection or contamination, is demonstrated. These results suggest that natural products of plants may be a promising and underappreciated reservoir to circumvent the existing antibiotic resistance.

## Graphical abstract

Isopentenylated flavonoids such as *α*‐mangostin and isobavachalcone are efficacious against multidrug‐resistant Gram‐positive and Gram‐negative pathogens with permeabilized outer membrane. Both *α*‐mangostin and isobavachalcone display rapid bactericidal activities through distinctive modes of action by targeting bacterial membrane phospholipids. Antibacterial agents targeting membrane exert fast time‐killing dynamics with less potential to develop resistance. Targeting membrane is an alternative strategy for antibacterial therapy.

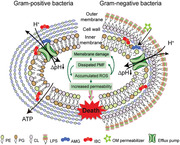

## 1 Introduction

Antibiotics are definitely the cornerstone of modern medical system.^[^ ^1^ ^]^ Emergence of multidrug‐resistant (MDR) bacteria at alarming rates is constantly paralyzing the health system worldwide.^[^ ^2^, ^3^, ^4^, ^5^ ^]^ Development of antibiotics with distinctive mechanisms is vital to win such arms race. Thus, novel efficient antibacterial agents and alternative strategies are urgently required to fill the void of antibiotic discovery and development. Compared to the synthetic chemotherapeutic drugs and other potential approaches,^[^ ^6^, ^7^, ^8^ ^]^ natural antibacterial agents possess the advances in accessibility,^[^ ^9^, ^10^ ^]^ structural diversity,^[^ ^11^ ^]^ robust activity,^[^ ^12^ ^]^ and distinct modes of action.^[^ ^13^ ^]^ Natural antibacterial agents and the analogues still dominate the multiple classes of antibiotics routinely used in clinic, including *β*‐lactams, tetracyclines, aminoglycosides, and polypeptides. The tremendous chemo‐diversity of natural products provides versatile scaffolds for antibiotic discovery, however, such natural compounds were largely screened from soil‐dwelling microorganisms based on Waksman platform.^[^ ^1^ ^]^ Although recent advances in biotechnologies make it possible to continually discover new antibiotics from untraditional terrestrial microbes^[^ ^6^, ^9^, ^14^, ^15^, ^16^ ^]^ and drug repurposing,^[^ ^17^, ^18^, ^19^ ^]^ the dramatically increased rediscovery rate of known antibiotics portends the declining number of drugs introduced to the clinic,^[^ ^13^ ^]^ lagging far behind the prompt dissemination of MDR pathogens globally. Therefore, the unexplored sources have been extended to mine new antibiotics from humans,^[^ ^7^, ^20^, ^21^ ^]^ insects,^[^ ^22^ ^]^ and other sources.^[^ ^11^, ^23^, ^24^ ^]^

Plants account for the most biomass on our planet.^[^ ^25^ ^]^ The discovery of bioactive molecules of plant origins has contributed to great progresses in drug development for various purposes.^[^ ^26^, ^27^, ^28^ ^]^ For instance, the chemical repertoire of herbs exhibits enormous natural compounds for the treatments of infection, particularly quinine and artemisinin against malaria.^[^ ^29^ ^]^ Lacking mammalian immune systems, plants evolutionarily optimize privileged drug‐like molecules to combat bacterial diseases.^[^ ^30^ ^]^ The largely untapped chemical diversity of traditional medicinal herbs has been historically neglected since the golden era of antibiotic discovery. Recently, by analyzing more than 183 natural products with antibacterial activity from plants, researchers found that plant natural products represent a promising source of antibacterial lead compounds that could help fill the drug discovery pipeline in response to the growing antibiotic resistance.^[^ ^31^ ^]^

**Figure 1:**
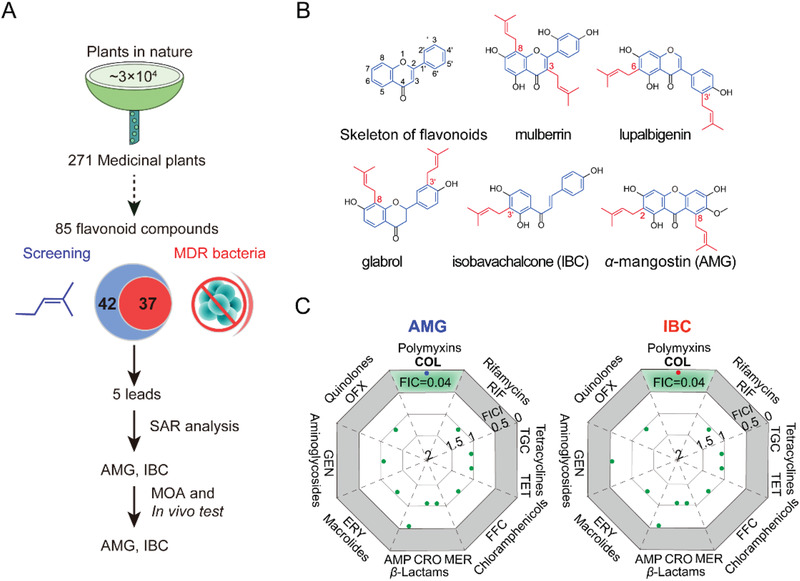
AMG and IBC show robust antibacterial activity against Gram‐positive bacteria and reverse colistin resistance in Gram‐negative bacteria. A) The scheme of screening antibacterial compounds in this study. 85 flavonoid compounds derived from 271 medicinal plants were collected and the antibacterial activities against Gram‐positive and Gram‐negative bacteria were determined by the broth micro‐dilution method according to the CLSI 2021 guideline. The SAR was analyzed and potent antibiotic candidates were chosen. Finally, the mode of action (MOA) and efficacy in vivo were clarified. B) Chemical structures of the skeleton of flavonoids and the five lead compounds; the skeleton of flavonoids was marked in blue and the isopentenyl group was in red. C) Synergy of AMG or IBC with different classes of antibiotics against *E. coli* B2. FICI were determined by chequerboard microdilution assays. Synergy is defined as a FICI ≤ 0.5. AMP, ampicillin; CRO, ceftriaxone; MER, meropenem; ERY, erythromycin; GEN, gentamicin; OFX, ofloxacin; COL, colistin; RIF, rifampicin; TGC, tigecycline; TET, tetracycline; and FFC, florfenicol. Data represent two biological replicates.

We reasoned that an alternative approach is to explore the untapped chemo‐diversity of plants for antibiotic discovery against MDR pathogens. Plants are abundant in a diverse group of polyphenolic flavonoids.^[^ ^32^, ^33^ ^]^ The ubiquitous flavonoids in medicinal herbs, accord for the multifaceted use of pharmaceutical, nutritional, and agrochemical interests.^[^ ^13^, ^34^, ^35^, ^36^, ^37^ ^]^ In this work, structure–activity relationship (SAR) analysis of flavonoids derived from 271 kinds of medicinal plants showed that certain isopentenylated flavonoids such as *α*‐mangostin (AMG) and isobavachalcone (IBC) were efficacious against antibiotic‐resistant priority Gram‐positive and Gram‐negative pathogens with permeabilized outer membrane (**Figure** **1**A). Mechanistic studies revealed that both AMG and IBC display rapid bactericidal activities through distinctive modes of action. Lastly, such flavonoids were further demonstrated in four models of MDR bacterial infection/contamination. Our findings suggest that structurally distinct antibacterial botanic molecules are an untapped source for discovering novel classes of drugs to circumvent the increasing prevalence of antibiotic resistance.

## 2 Results

### 2.1 Screening of Flavonoids and the SAR Analysis

Flavonoids share a common skeleton with the 15‐carbon 2‐phenyl‐chromone core (C6—C3—C6 system, Figure 1B),^[^ ^38^ ^]^ which are further divided into nearly 20 groups.^[^ ^33^ ^]^ Antibacterial activities of flavonoids have been controversially reported;^[^ ^13^, ^39^, ^40^ ^]^ it remains unclear to what extent the side‐chain modification of flavonoids affects the activity. Since the presence of prenyl‐moiety can sharply increase the lipophilicity of natural products,^[^ ^41^, ^42^ ^]^ we hypothesized whether and how the prenylation modulates the activity of flavonoids through attachment to bacterial membrane. The largely unexplored bacterial membrane remains an intriguing potential target for antibiotic discovery.^[^ ^18^, ^43^, ^44^ ^]^ Therefore, 85 typical flavonoids including 23 flavones, 20 isoflavones, 13 flavanones, 10 chalcones, and 19 xanthones, were collected for antibacterial tests, according to the CLSI 2021 guideline.^[^ ^45^ ^]^ Interestingly, more than 40% compounds (37/85) were active against *Staphylococcus aureus* (*S. aureus*) ATCC 29213 and methicillin‐resistant *S. aureus* (MRSA T144), with the minimum inhibitory concentrations (MICs) ranging from 1 to 8 µg mL^−1^ (Table S2, Supporting Information). Although no direct antibacterial effects on *Escherichia coli* (*E. coli*) were observed, 35.2% compounds (30/85) restored the susceptibility of the model MDR *E. coli* B2 isolate^[^ ^8^ ^]^ in the presence of colistin.

Interestingly, all the flavonoids that showed antibacterial activity against MRSA T144 are prenylated (37/37) (Table S2, Supporting Information). The number of prenyl groups attached to the backbone affects the activity as well, based on SAR analysis. Compared to monoprenyl flavonoids (33.3%, 15/45) and triprenyl groups modified ones (50%, 4/8), the prenylation with two groups (69.2%, 18/26) dominates the active prenylated flavonoids. Generally, we found that the position of prenylation mainly at C8 (44.4%, 16/37), C6 (30.6%, 11/37), C3 (22.2%, 8/37), and C3’ (18.9%, 7/37) of the phenolic skeleton was a prerequisite for the antibacterial activity, which is in accordance with previous studies.^[^ ^46^ ^]^ Taken together, five lead compounds: mulberrin, lupalbigenin, glabrol, AMG, and IBC (Figure 1B) were selected from the major subgroups of flavonoids for subsequent tests of antibacterial generality and mechanistic study.

### 2.2 AMG and IBC are Potent Antibiotic Candidates

All leads are active against *S. aureus* ATCC 29213, MRSA T144, and VRE_fm_ CAU 369 with MIC less than 4 µg mL^−1^ (Table S3, Supporting Information). Notably, a comparison among the five leads showed that AMG and IBC under 8 µg mL^−1^ dramatically restored the activity of colistin against MDR *E. coli* B2 isolate, with the decreased concentration of colistin from 8 to 0.0625 µg mL^−1^ (Table S4, Supporting Information). To further evaluate the potential of AMG and IBC, MIC_50_ and MIC_90_ against MRSA and VRE were determined. The MIC_50_ and MIC_90_ against MRSA and VRE were 0.5 and 4–8 µg mL^−1^ for AMG and IBC, respectively (**Table** **1**; Tables S5 and S6, Supporting Information). Both AMG and IBC display similar efficiency to vancomycin. These results indicate that AMG and IBC are potent antibiotic candidates to combat MDR bacteria, practically against Gram‐positive pathogens.

**Table 1:** Antibacterial activities of AMG and IBC

	MIC (µg mL^−1^)		
Organism	AMG	IBC	VAN
*S. aureus* ATCC 29213	1	4	1
*S. aureus* T144 (MRSA)	1	4	0.5
*S. aureus* 65322 (MRSA)	0.5	4	1
*E. faecium* CAU 382 (VRE_fm_)	0.5	8	>128
VRE_fm_ CAU 383	0.5	4	>128
VRE_fm_ CAU369	2	1	>128
*E. coli* ATCC 25922	>128	>128	>128
*E. coli* B2	>128	>128	>128
MRSA (*n* = 23)			
MIC_50_	0.5	4	1
MIC_90_	0.5	4	2
VRE (*n* = 50)			
MIC_50_	0.5	8	>128
MIC_90_	0.5	8	>128

MRSA, methicillin‐resistant *S. aureus*; VRE_fm_, vancomycin‐resistant *Enterococcus faecium*; AMG, *α*‐Mangostin; IBC, isobavachalcone; VAN, vancomycin. *E. coli* B2, MDR clinical isolate carrying 25 antibiotic resistance genes.

Given the potentiation of AMG and IBC on colistin against *E. coli* B2, we extended their effects on multiple classes of antibiotics including tetracycline, ofloxacin, rifampicin, and ampicillin. We found that 2–8 µg mL^−1^ AMG and IBC exclusively reversed colistin resistance in *E. coli* B2 (FICI = 0.04, Figure 1C), with 16‐ to 128‐fold decrease of the MICs of colistin from 8 µg mL^−1^ to 0.0625–0.5 µg mL^−1^ (Figure S1, Supporting Information). Meanwhile, AMG and IBC enhanced the activity of colistin against colistin sensitive isolates with the MICs decreasing from 0.125–0.25 µg mL^−1^ to 0.031–0.002 µg mL^−1^ as well (Figure S2, Supporting Information). To investigate the generality of such combination, we subsequently obtained the values of FIC on various Gram‐negative bacterial species.^[^ ^8^ ^]^ AMG and IBC fully restored the sensitivity of all these ten species of Gram‐negative bacteria to colistin carrying mobile colistin resistant gene *mcr‐1* (FICI < 0.1, Figure S3A, Supporting Information). To validate the potentiation, we further assessed such combinations on 87 multi‐drug resistant clinical *E. coli* isolates carrying *mcr‐1*.^[^ ^8^, ^47^ ^]^ There is more than 90% inhibition of bacterial growth (Figure S3B,C, Supporting Information), under the treatments with the combinations of either 2 µg mL^−1^ AMG or 4 µg mL^−1^ IBC with 0.5 µg mL^−1^ colistin. The potentiation of either AMG or IBC with colistin seems efficient than the first reported colistin adjuvant pentamidine^[^ ^48^ ^]^ and is comparable to that of the broad‐spectrum antibiotic adjuvant SLAP‐S25.^[^ ^8^ ^]^

In addition, we found AMG showed low hemolysis (25.86%) to sheep red blood cells at 32 µg mL^−1^ and IBC showed negligible hemolysis (0.73%) even at 128 µg mL^−1^ (Figure S4, Supporting Information). No de novo resistance to AMG or IBC was observed during 30‐day serial passage of *S. aureus* ATCC 29213 (Figure S4B, Supporting Information). These results indicate that both AMG and IBC are potent antibiotic candidates against MRSA and VRE, as well as promising adjuvants of colistin against MDR Gram‐negative pathogens.

### 2.3 AMG and IBC Target Bacterial Inner Membrane

**Figure 2:**
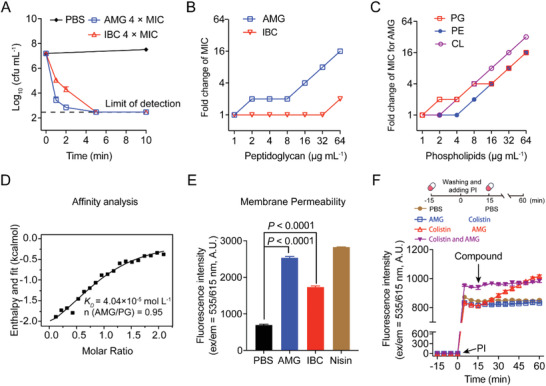
AMG and IBC exert antibacterial effects through membranes. A) Time‐kill curves of AMG and IBC against *S. aureus* ATCC 29213 in the exponential phase at 37 °C. 4 × MIC of AMG, IBC killed the bacteria rapidly. B) Exogenous addition of peptidoglycan from *S. aureus* decreased the antibacterial activities of AMG and IBC determined by chequerboard microdilution assays. The concentration of peptidoglycan was in the range of 0 to 64 µg mL^−1^. C) Exogenous addition of phosphatidylglycerol (PG), phosphatidylethanolamine (PE), and cardiolipin (CL) abolished the antibacterial activity of AMG against *S. aureus* ATCC 29213, determined by chequerboard microdilution assays. The concentrations of phospholipids were in the range of 0 to 64 µg mL^−1^. D) Isothermal titration calorimetry (ITC) analysis of the interaction between PG and AMG. Thermodynamic parameters were calculated, including equilibrium dissociation constant (*K*_D_ = 4.04 × 10^−5^ mol L^−1^), number of binding sites (*n* = 0.95), molar binding enthalpy (*ΔH* = −11.87 kJ mol^−1^), and molar binding entropy (*ΔS* = 44.28 J mol^−1^ K^−1^). E) Increased membrane permeability after treatment of AMG or IBC at 4 × MIC. The membrane permeability was determined by propidium iodide (PI) with the excitation/emission wavelength at 535 nm/615 nm. F) The dynamic curves of the permeability of inner membrane probed with PI for *E. coli* B2, under the treatments of AMG, and colistin and both thereof. *E. coli* B2 were incubated with different antibacterial drugs at 37 °C for 15 min. After washing for three times, PI was added and the fluorescence was determined for 15 min. Then the other drugs were added. The fluorescence was measured with the excitation wavelength at 535 nm and emission wavelength at 615 nm. The concentrations of AMG and colistin were 2 and 0.25 µg mL^−1^, respectively. All experiments in A, E, and F were performed as three biologically independent experiments, and the mean ± s.d. is shown, *n* = 3. Data in (B,C) represent two biological replicates. *P* values in E were determined using unpaired student's *t*‐test.

We first explored the time‐kill dynamics to investigate the antibacterial mechanisms of AMG and IBC. Surprisingly, both AMG and IBC revealed rapid bactericidal activities against *S. aureus* in a dose dependent manner (Figure S5A,B, Supporting Information). Both could promptly reduce the viable bacteria below the limit of detection at high levels in 1 min, and AMG showed robust activity than IBC at low levels (**Figure** **2**A). It may account for no observed mutant during resistance development assay, and denote that such hydrophobic prenylated flavonoids probably target bacterial membrane. Therefore, we determined the effects of various components of bacterial wall and membrane on the activity of AMG and IBC against *S. aureus*. Compared to the slight reduction of MICs in the presence of peptidoglycan at low levels (Figure 2B), a major component in cell wall, exogenous addition of bacterial phospholipids in the cell membrane diminished the activity of AMG and IBC in a dose‐dependent manner, particularly for AMG (Figure 2C; and Figure S5C–E, Supporting Information). Consistently, the affinity between AMG and phosphatidylglycerol (PG) (*K*_D_ = 4.04 × 10^−5^ mol L^−1^) is approximately tenfold higher than that of IBC (*K*_D_ = 3.04 × 10^−4^ mol L^−1^) (Figure 2D; and Figure S5F,G, Supporting Information), based on isothermal titration calorimetry (ITC) analysis. As a consequence, the increase of membrane permeability was observed in *S. aureus* treated with either AMG or IBC (Figure 2E). Collectively, the results suggest that the interference with bacterial cell membranes by prenylated flavonoids may be responsible for their robust bactericidal activities against Gram‐positive pathogens.

With regard to the same components of phospholipids in bacterial inner membrane, we denoted that the intact permeability barrier of lipopolysaccharide (LPS) prevents hydrophobic prenylflavonoids from attaching to the inner membrane in Gram‐negative bacteria. It is consistent with the broad‐spectrum and exclusive potentiation of AMG/IBC in combination with antibiotics tested against Gram‐negative pathogens (Figure 1). To elucidate the role of LPS, we first assessed the addition of exogenous LPS or cations, because divalent cations particularly Mg^2+^ are required for the integrity of outer membrane.^[^ ^8^ ^]^ High levels of LPS and divalent cations have neglectful influence on the activity of either AMG or IBC (Figure S6A–C, Supporting Information), supporting that Gram‐negative bacteria are ineffective to such leads highly due to the physical presence of LPS. Thus, we determined the activity of AMG or IBC in the mutants with defective LPS. Notably, the mutants of *Klebsiella pneumoniae* (*K. pneumoniae*) and *Acinetobacter baumannii* (*A. baumannii*) were sensitive to AMG or IBC, whereas they changed the susceptibility to colistin accordingly (Table S7, Supporting Information). For instance, the MIC of AMG dramatically decreased from more than 128 µg mL^−1^ in the wild‐type *A. baumannii* to 0.125 µg mL^−1^ in the mutant with deleted LPS,^[^ ^49^ ^]^ without synergy in the presence of colistin (Figure S6D,E, Supporting Information). These findings confirm that the synergetic mechanism is caused by the increase of permeability of the outer membrane, facilitating the attachment of AMG and IBC to the inner membrane. Constantly, the colistin analogue polymyxin B nonapeptide (PMBN), which solely increases the permeability of the outer membrane,^[^ ^50^ ^]^ displayed comparable synergy with either AMG or IBC against *E. coli* (Figure S6F,G, Supporting Information). To further decipher the mechanism of the combinations, we recorded the permeability dynamics of inner membrane in *E. coli* B2, under the treatments of AMG or IBC, and colistin and both thereof (Figure 2F; and Figure S6H,I, Supporting Information). We observed steady increase of fluorescent intensity upon the addition of AMG (Figure 2F). Altogether, these results suggest that AMG and IBC are effective to kill Gram‐negative bacteria through paralyzing the cytoplasmic membrane, when the outer membrane is permeabilized.

### 2.4 Mechanism of AMG and IBC for Killing Bacteria

**Figure 3:**
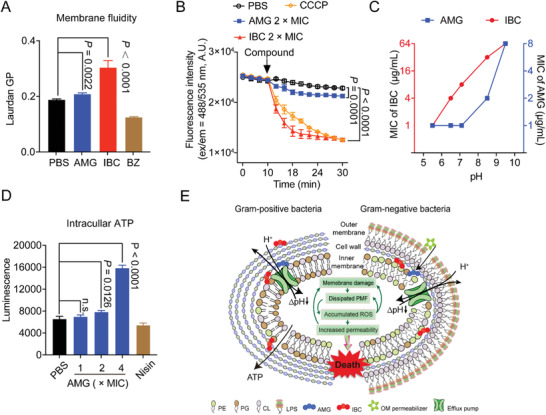
AMG and IBC exerts antibacterial effects through membranes. A) The fluidity of membrane was decreased for *S. aureus* ATCC 29213 after treatment of AMG and IBC for 30 min. 50 mmol L^−1^ of benzyl alcohol (BZ) was used as the positive control. B) Dissipated *Δ*pH by AMG and IBC in *S. aureus* ATCC 29213. Exponential *S. aureus* ATCC 29213 was incubated with pH fluorescence probe BCECF‐AM. After washing three times, different concentrations of AMG and IBC were added and the intracellular pH was determined by measuring the fluorescence intensity with the excitation/emission wavelength at 488 nm/535 nm. C) Decreased antibacterial activity of AMG and IBC in alkaline medium. D) Increased levels of intracellular ATP in *S. aureus* ATCC 29213 after treatment of AMG for 10 min. E) Mechanism of actions of AMG and IBC in Gram‐positive and Gram‐negative bacteria, respectively. All experiments in (A,B,D) were performed as three biologically independent experiments, data presented as mean ± s.d, *n* = 3. *P*‐values in (A,B) were calculated using unpaired Student's *t*‐test. *P*‐values in (D) were calculated using non‐parametric one‐way ANOVA.

The biophysical integrity and function of the inner membrane is of vital importance for bacterial growth and survival.^[^ ^51^, ^52^ ^]^ Recently, pioneering works demonstrate bacterial inner membrane is a promising target for antibiotic discovery.^[^ ^21^, ^43^ ^]^ To dissect the underlying mechanisms of AMG and IBC, we focused on the subsequent dysfunction of membrane using *S. aureus* ATCC 29213 as a model. We first measured the membrane fluidity by Laurdan probe,^[^ ^53^ ^]^ and found significant decrease of fluidity in the presence of AMG or IBC (**Figure** **3**A). It is in agreement with that, the change of membrane rigidity disrupts bacterial homeostasis resulting in fundamental metabolic disorders including the dissipation of proton motive force (PMF).^[^ ^54^ ^]^ Hence, we evaluated the *Δ*pH, a key component of the proton motive force,^[^ ^55^ ^]^ in *S. aureus* using the fluorescent probe BCECF‐AM. Compared to AMG, the *Δ*pH significantly dissipated in *S. aureus* treated with 2 × MIC IBC in 5 min (Figure 3B), agreeing with the prompt bactericidal effect. Furthermore, we determined the antibacterial activity in MHB medium with the pH ranging from 5.5 to 9.0, because the pH shift in the extracellular microenvironment leads to the decrease of *Δ*pH. IBC showed better antibacterial activity than AMG under acidic conditions (Figure 3C), where the PMF is mainly maintained by *Δ*pH. PMF is modulated by a delicate compensation mechanism that dissipation in either component of *Δ*pH or membrane potential (*Δψ*) is compensated by a counteractive increment in the other.^[^ ^56^ ^]^

Subsequently, we measured the *Δψ* by a fluorescent dye 3, 3‐dipropylthiadicarbocyanine iodide [DiSC_3_(5)],^[^ ^57^ ^]^ and we found that AMG bound to the dye and quenched the fluorescence (Figure S7A, Supporting Information). To clarify the disruption of *Δ*pH, we determined the activity of AMG or IBC with kanamycin. It consists of the synergy of kanamycin in combination with either AMG or IBC against *S. aureus* (Figure S7B, Supporting Information), since the uptake of aminoglycoside antibiotics is *Δψ* dependent.^[^ ^58^ ^]^ Correspondingly, AMG or IBC shows no synergy with tetracycline, since its accumulation is *Δ*pH dependent (Figure S7C, Supporting Information). As a consequence, disrupted membrane homeostasis always contributes to the accumulation of reactive oxygen species (ROS).^[^ ^59^ ^]^ Like many bactericidal antibiotics, the activity of IBC is dependent on the accumulation of intracellular ROS (Figure S7E, Supporting Information), correspondingly aggravating membrane damage. Interestingly, AMG has no effect on ROS (Figure S7D, Supporting Information). Interestingly, we observed the increase of ATP levels in an AMG‐dose dependent manner [Figure 3D; and Figure S7F (Supporting Information)], whereas IBC could slightly stimulate the production of ATP (Figure S7G,H, Supporting Information). Such results are consistent with the previous studies that bactericidal antibiotics are associated with accelerated respiration.^[^ ^60^ ^]^ Similarly, we found the same trends of membrane permeability, *Δ*pH, and ROS, whereas there was increase of membrane fluidity in *ΔwaaC K. pneumoniae* ATCC 43816 under the treatment of AMG or IBC (Figure S8, Supporting Information). These findings indicate that the modes of action of prenylflavonoids such as AMG and IBC share distinctive modes of action, which are promising candidates to circumvent the existing resistance mechanisms and avoid cross resistance.

Taken together, our results suggest that the prenylation of flavonoids at appropriate positions plays a critical role in the antibacterial activity, particularly for MDR pathogens. Both lead compounds AMG and IBC target bacterial phospholipids, collapsing the membrane homeostasis in Gram‐positive bacteria, and show synergy combined with the permeabilizer of outer membrane against Gram‐positive bacteria (Figure 3E).

### 2.5 Efficacy of AMG and IBC in Models

**Figure 4:**
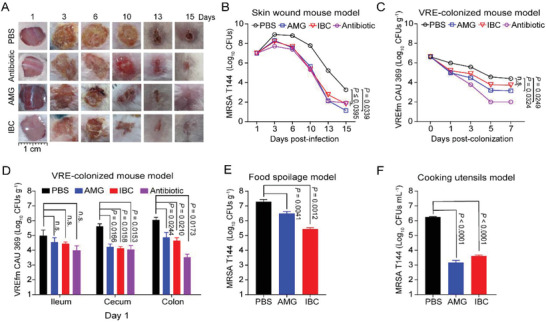
AMG and IBC exerted greatly therapeutic potential in in vivo and in vitro models. A) Mouse skin wound infection models. Representative photographs of the wounds under the treatment of AMG (2 mg kg^−1^), IBC (8 mg kg^−1^) or vancomycin (1mg kg^−1^) (*n* = 18 in each group). The morphology of the wounds under the treatment of AMG or IBC displayed comparable trends compared with antibiotic (vancomycin). B) AMG and IBC reduced the bacterial loads in skin wound infections. C) VRE‐colonized mouse model. AMG (5 mg kg^−1^) and IBC (20 mg kg^−1^) rapidly decreased fecal number of VRE_fm_ CAU369 (1.0 × 10^9^ CFUs) after VRE colonization. Antibiotic (tiamulin, 5 mg kg^−1^) was used as a positive control. D) VRE‐colonized mouse model. AMG (5 mg kg^−1^) and IBC (20 mg kg^−1^) decreased the bacterial loads of VRE_fm_ CAU369 in ileum, cecum, and colon after treatment for 1 day. Antibiotic (tiamulin, 5 mg kg^−1^) was used as a positive control. E) Food spoilage model. Treatments with AMG (2 mg kg^−1^) and IBC (4 mg kg^−1^) reduced bacterial numbers in pork infected with MRSA T144. F) Cooking utensils model. Either AMG or IBC dramatically decreased the number of MRSA T144 on the surface of contaminated cooking utensils. Plastic lunch boxes were contaminated with MRSA T144 (6.0 × 10^6^ CFUs) for 30 min. After treatment of AMG (0.1 µg) or IBC (0.4 µg) for 2 min, the bacteria on the lunch boxes was calculated by chromogenic agar plate assay. Data presented as mean ± s.d, *n* = 3. *P*‐values were calculated using one‐way ANOVA.

The encouraging antibacterial activity of AMG and IBC in vitro inspired us to further investigate their therapeutic potential in infectious models. We constructed models of *S. aureus* wound infection and gut colonization of VRE in mice, using single administration of AMG or IBC. The morphology and size of wound displayed comparable trends under the treatment of 2 mg mL^−1^ AMG, 8 mg mL^−1^ IBC, and 1 mg mL^−1^ vancomycin (**Figure** **4**A; and Figure S9A, Supporting Information). Consistently, there was more than tenfold reduction of bacterial numbers from the infectious sites (Figure 4B). Furthermore, VRE_fm_ CAU 369 (1.0 × 10^9^ colony‐forming units (CFUs)) was colonized into the gastrointestinal tract of mice by intragastric administration. Compared to the moist and soft feces in the infected group, the fecal morphology was restored in the mice treated with either AMG or IBC (Figure S9B, Supporting Information). The reduction of colonization was supported by the rapidly decreased fecal number of *Enterococcus. faecium* (Figure 4C), which is in agreement with the decline of colonized *E. faecium* in gut (Figure 4D; and Figure S9C, Supporting Information). These results demonstrated the promising pharmacological efficacy of AMG and IBC in vivo. Given the widely used plant extracts in food industry, we tested the antiseptic and disinfectant capabilities on food associated materials. We found that AMG (2 mg kg^−1^) and IBC (4 mg kg^−1^) could alleviate the spoilage in pork caused by *S. aureus* (6 × 10^3^ CFUs per part). The numbers of *S. aureus* significantly reduced (Figure 4E), with well retained quality and odor of the meat (Figure S9D, Supporting Information). Additionally, we evaluated the disinfection effect of AMG and IBC on plastic lunch boxes contaminated with *S. aureus* (6.0 × 10^6^ CFUs mL^−1^). Either 2 µg mL^−1^ AMG or 8 µg mL^−1^ IBC dramatically decreased the number of *S. aureus* on the surface of contaminated boxes in 2 min (Figure 4F), with no detectable bacteria in 10 min (Figure S9E, Supporting Information). The results indicate that such natural compounds may be alternatively used to reduce the incidence of foodborne bacterial infections and poisoning.

## 3 Discussion

Although bioactive molecules of plant origins have been utilized for various purposes,^[^ ^25^, ^26^ ^]^ the application to combat MDR bacteria and restore the activity of clinic antibiotics has been ignored for a long time. We evaluated 85 flavonoids derived from 271 kinds of medicinal plants and found that more than 40% compounds showed the antibacterial activity against MDR bacteria including MRSA and VRE, and 35.2% compounds restored the susceptibility of a model MDR *E. coli* B2 isolate in the presence of colistin (Table S2, Supporting Information). Based on the SAR analysis, we found that prenylation with two groups attached to the backbone and the position of prenylation of the phenolic skeleton was a prerequisite for the antibacterial activity, which is in accordance with previous studies.^[^ ^46^ ^]^ Among the active substances, five leads show comparable activity to vancomycin (Table S3, Supporting Information) and other candidates such as malacidins.^[^ ^21^ ^]^ Besides, the synergy of AMG or IBC with colistin was comparable to colistin adjuvant pentamidine^[^ ^47^ ^]^ and SLAP‐S25.^[^ ^8^ ^]^ These results demonstrated that the plants and prenylated flavonoids from plants are promising resources to develop antibiotic candidates against MDR bacteria.

The biophysical integrity and function of the inner membrane is of vital importance for bacterial growth and survival.^[^ ^18^, ^43^ ^]^ The membrane is a promising target for screening antibiotic candidates and antibacterial agents targeting the bacterial membrane have therapeutic potential.^[^ ^13^, ^53^, ^61^ ^]^ For example, the broad‐spectrum adjuvant SLAP‐S25 exerted the activity by targeting the PG in bacterial membrane. In our study, two leads, AMG and IBC, could target nearly all kinds of phospholipids in bacterial membrane with the faster time‐killing dynamics than nearly all of the antibiotics used in clinic including vancomycin (Figure 2A; and Figure S5A,B, Supporting Information). Such membrane‐disrupting agents have less potential to develop antibiotic resistance, as bacterial membrane can hardly change without loss of function. Notably, although exerting antibacterial activity rapidly for both AMG and IBC, the following mechanisms for AMG and IBC are distinct, avoiding the evolution of cross resistance.^[^ ^61^ ^]^ Compared with chemically synthesized SLAP‐S25 as a broad‐spectrum antibiotic adjuvant, naturally ubiquitous AMG and IBC are easily accessible with direct bactericidal activity against MDR bacteria. Notably, SLAP‐S25 with high affinity to PG can also guide the structural optimization of AMG and IBC through thermodynamic profiling and the synthesis of flavonoid‐based mimetics to improve their binding selectivity.^[^ ^62^, ^63^ ^]^

Although we have demonstrated the mechanism of AMG and IBC initially, AMG is bactericidal and is independent of the ROS, which are not accordant with previous studies.^[^ ^59^ ^]^ Besides, the mechanism for better activity of AMG than IBC could help us to discover and screen potent antibacterial candidates. The determinants for AMG and IBC to kill bacteria still need further studies and pharmacodynamical behaviors need to be optimized in the future.

In conclusion, we show that prenylated flavonoids from plants exhibit robust antibacterial activities against clinically important pathogens including Gram‐positive pathogens MRSA and VRE, and can serve as an antibiotic adjuvant to potentiate the efficacy of colistin against diverse Gram‐negative pathogens. Both AMG and IBC are promising candidates for the development of novel antibiotic agents to combat MDR bacterial pathogens associated infections.

## 4 Experimental Section

### Antibacterial Tests

Minimum inhibitory concentrations (MICs) of potential lead candidates and other antibiotics were determined by the broth micro‐dilution method, according to the CLSI 2021 guideline.^[^ ^45^ ^]^ Briefly, the potential lead candidates or other antibiotics were twofold diluted in Mueller–Hinton Broth (MHB, Beijing Land Bridge Technology) and mixed with an equal volume of bacterial suspensions in MHB containing approximately 1.5 × 10^6^ CFUs mL^−1^ in a clear UV‐sterilized 96‐well microtiter plate (Corning). The lowest concentrations of antibiotics with no visible growth of bacteria were the MICs after incubation at 37 °C for 18 h. The pH of the medium was adjusted to 5–10 by HCl or NaOH if needed.

### Checkerboard Assays

Fractional inhibitory concentrations (FICs) were determined by checkerboard assays as described previously.^[^ ^8^ ^]^ First, 100 µL MHB medium was dispensed into a 96‐well plate. Flavonoids (100 µL) were added to the last row, then diluted along the ordinate. Subsequently, colistin was added to the first column and diluted along the abscissa. Finally, in addition to the negative control, 100 µL bacterial suspensions ≈ 1 × 10^6^ CFUs mL^−1^ were added. After culturing at 37 °C for 18 h, the MICs were recorded as the lowest concentration of drug inhibiting visible growth. The synergistic effect was determined by calculating FIC. The FIC was calculated according to the formula as follows:

(1) $$\mathrm{FIC}\;\mathrm{index}=\mathrm{MI}C_{ab}/\mathrm{MI}C_{a}+\mathrm{MI}C_{ba}/\mathrm{MI}C_{b}=\mathrm{FI}C_{a}+\mathrm{FI}C_{b}$$

MIC*_a_* is the MIC of compound *a* alone; MIC*_ab_* is the MIC of compound *a* in combination with compound *b*; MIC*_b_* is the MIC of compound *b* alone; MIC*_ba_* is the MIC of compound *b* in combination with compound *a*; FIC*_a_* is the FIC of compound *a*; FIC*_b_* is the FIC of compound *b*. The synergy or additive was defined according to standard criteria (FICI ≤ 0.5 was defined as synergistic; 0.5 < FICI ≤ 1 was defined as additive; 1 < FICI ≤ 4 was defined as indifference; FICI > 4 was defined as antagonism).

### Growth Curves of Bacteria

*E. coli* B2 was cultured in 1 mL of brain heart infusion (BHI, Beijing Land Bridge Technology) broth for approximately 16 h at 37 °C with shaking at 200 rpm. The overnight cultures were diluted 1:100 in MHB and adjusted to approximately 1 × 10^6^ CFUs mL^−1^. Different concentrations of AMG, IBC, colistin, or the combination of colistin with AMG or IBC were added into a 96‐well microplate and mixed with an equal volume of bacterial dilutions. Growth curves were established under the wavelength of OD_600 nm_ with an interval of 1 h at 37 °C, by Infinite M200 Microplate reader (Tecan).

### Time‐Kill Curves

*S. aureus* ATCC 29213 cultured to exponential phase at 37 °C with shaking at 200 rpm were diluted in MHB to a desired concentration at 10^6^ to 10^7^ CFU mL^−1^. Different concentrations of AMG or IBC (2 ×, 4 ×, and 10 × MIC) were added and cultured at 37 °C with shaking at 200 rpm. 100 µL aliquots were removed after culturing for 2, 5, 10, 15, and 20 min, and then were tenfold serially diluted and plated on TSA plates to calculate the colony‐forming units (CFUs) after incubation at 37 °C for 24 h.

### Antibacterial Activity of the Mixtures of AMG or IBC with Lipids

Different phospholipids including phosphatidylglycerol (PG, Sigma–Aldrich, 841188P, ≥ 99%), phosphatidyl–ethanolamine (PE, Sigma–Aldrich, 840027P, ≥ 99%), or cardiolipin (CL, Sigma–Aldrich, 841199P, ≥ 99%) was dissolved in methanol. Effects of various phospholipids (0 to 128 µg mL^−1^) on the antibacterial activity of AMG or IBC in MHB medium were determined using the chequerboard microdilution assay.

### Isothermal Titration Calorimetry (ITC) Assays

To evaluate the interaction between 1‐palmitoyl‐2‐oleoyl‐*sn*‐glycero‐3‐phospho‐(1'‐rac‐glycerol) sodium salt (POPG, Sigma–Aldrich, 63371, ≥98%) and AMG or IBC, calorimetric experiment was performed by MicroCal ITC (Malvern Panalytical) at 25 °C. PG dissolved in HEPES (20 mmol L^−1^, pH 7.0) was sequentially injected into the calorimetric cells filled with AMG or IBC which was dissolved in the same buffer, and the injection was repeated 20 times with an equilibrium interval of 200 s. The processed data was used, the relative software with the instrument calculating the equilibrium dissociation constant (*K*_D_), stoichiometry (*n*), the changes of enthalpy (*ΔH*) and entropy (*ΔS*).

### Constructions of LPS Knockout Bacteria

*WaaC*‐deficient strain of *K. pneumoniae* ATCC 43816 was constructed by CRISPR‐Cas9 according to previous study.^[^ ^64^ ^]^ *A. baumannii* 7‐2 LPS‐ (LPS deficient) was obtained as described in literature.^[^ ^65^ ^]^

### Membrane Integrity Assays

Overnight cultures of *S. aureus* ATCC 29213 or *K. pneumonia* ATCC 43816 Δ*waaC* were centrifuged and resuspended with 0.01 mol L^−1^ PBS (pH 7.4) to adjust bacterial suspensions to approximately an OD_600 nm_ of 0.5, followed by the addition of AMG or IBC (final concentrations of 0, 1, 2, 4, 8, 16, and 32 µg mL^−1^). The bacterial suspensions were incubated at 37 °C for 20 min without light, then probed with propidium iodide (PI, Thermo Scientific, P1304MP) at a final concentration of 10 nmol L^−1^. After incubation for 20 min, the fluorescence values were determined by Infinite M200 Microplate reader (Tecan) with excitation wavelength at 535 nm and emission wavelength at 615 nm. The dynamic curves of the permeability of inner membrane for *E. coli* B2 were probed with PI. In brief, overnight cultures of *E. coli* B2 were adjusted to approximately an OD_600 nm_ of 0.5 with 5 mmol L^−1^ HEPES and 5 mmol L^−1^ glucose buffer (pH = 7.2). *E. coli* B2 were incubated with different antibacterial drugs at 37 °C for 15 min. After washing for three times, PI was added and the fluorescence was determined for 15 min. Then, the other drugs were added. The fluorescence was measured with the excitation wavelength at 535 nm and emission wavelength at 615 nm.

### Membrane Fluidity Assays

Overnight cultures of *S. aureus* ATCC 29213 or *K. pneumonia* ATCC 43816 Δ*waaC* were grown in BHI for 6–8 h at 37 °C with shaking and resuspended with 0.01 mol L^−1^ PBS (pH 7.4) to adjust bacterial suspensions to approximately an OD_600 nm_ of 0.5. Then, *S. aureus* was stained by a final concentration of 10 nmol L^−1^ laurdan, and incubated at 37 °C for 10 min. Subsequently, bacterial suspensions were treated with 1 ×, 2 ×, and 4 × MIC concentrations of AMG, IBC, or benzyl alcohol for another 35 min. Lastly, the fluorescence was determined by the Infinite M200 Microplate reader (Tecan) with excitation wavelength at 350 nm and emission wavelength at 438 nm. Laurdan GP was calculated according to previous studies.

### *Δ*pH Measurement

*S. aureus* ATCC 29213 or *K. pneumonia* ATCC 43816 Δ*waaC* cultured overnight was washed with HEPES (5 mmol L^−1^, pH 7.0, plus 5 mmol L^−1^ glucose) and resuspended to obtain an OD_600 nm_ of 0.5. The *Δ*pH was determined by the pH‐sensitive fluorescence probe BCECF‐AM. Briefly, BCECF‐AM was added with final concentration of 10 µmol L^−1^ and incubated at 37 °C. Then 10 µL AMG or IBC solution (final concentrations of 1 ×, 2 ×, and 4 × MIC) were added and the fluorescence value was determined by Infinite M200 Microplate reader (Tecan), with the excitation wavelength at 488 nm and emission wavelength at 535 nm.

### ATP Determination

Extracellular and intracellular ATP levels of *S. aureus* ATCC 29213 were determined using an Enhanced ATP Assay Kit (Beyotime, catalogue no. S0027). *S. aureus* ATCC 29213 was cultured overnight at 37 °C with shaking at 200 rpm, subsequently washed and resuspended in 0.01 mol L^−1^ of PBS to obtain an OD_600 nm_ of 0.5. Then the bacterial suspensions were treated with various concentrations of AMG (1–4 µg mL^−1^) and of IBC (4–16 µg mL^−1^) for 10 min and then centrifuged at 10 000 rpm at 4 °C for 5 min, and the bacterial supernatant was collected for the extracellular ATP level determination. Meanwhile, the bacterial precipitates lysed by lysozyme and centrifuged could be used for measuring intracellular ATP level. The detecting solution was added to a 96‐well plate and incubated at room temperature for 5 min. The supernatants were added and mixed quickly, and determined using the Infinite M200 Microplate reader (Tecan) in the model of luminescence.

### Haemolytic Activity

Sterile defibrinated sheep blood cells were washed with PBS for three times, then 100 uL of 8% blood cells were added to 100 uL different concentrations of AMG (0 to 512 µg mL^−1^) or IBC (0 to 512 µg mL^−1^) in 96 well U‐bottom plate. Meanwhile, 0.2% Triton X‐100 and PBS were used as positive and negative control, respectively. After 1 h at 37 °C, cells were centrifuged at 3000 g for 10 min, then 100 µL supernatant was taken to determine its absorbance at 576 nm by Infinite M200 Microplate reader (Tecan).

### Resistance Development Studies

Overnight cultures of *S. aureus* ATCC 29213 were inoculated in fresh MHB containing 0.25, 0.5, 1, 2, and 4 × MIC of AMG or IBC. Oxacillin was used as a positive control. The bacterial cultures were then incubated at 37 °C under continuous shaking at 200 rpm for 24 h. Subsequently, the MIC of bacteria from the second‐highest concentrations with visible growth (OD_600 nm_ ≥ 0.3) was determined by broth microdilution in fresh MHB media containing different concentrations of AMG or IBC. The cultures were serially passaged for 30 days.

### Peptidoglycan, LPS, and Cationic Ion Assays

The effect of peptidoglycan, LPS, or different cations including NaCl, KCl, CaCl_2_, CuSO_4_, MgCl_2,_ and FeCl_3_ (Aladdin) on antimicrobial activity of AMG or IBC against *S. aureus* ATCC 29213 was evaluated using checkerboard assays.

### PMBN Assays

The synergistic effects of colistin analogue polymyxin B nonapeptide (PMBN) and AMG or IBC against *E. coli* ATCC 25922 were determined using the chequerboard microdilution assay.

### Competition Kinetics

Different concentrations of POPG (0–8 µg mL^−1^) were added to the mixture of 3, 3‐dipropylthiadicarbocyanine iodide DiSC_3_ (5) (1 µmol L^−1^) and AMG (4 µg mL^−1^) in 0.01 mol L^−1^ PBS (pH 7.4). Then, the fluorescence value of DiSC_3_ (5) was determined by Infinite M200 Microplate reader (Tecan), with excitation wavelength at 622 nm and emission wavelength at 670 nm.

### ROS Measurement

*S. aureus* ATCC 29213 or *K. pneumonia* ATCC 43816 Δ*waaC* cultured overnight was washed with 0.01 mol L^−1^ PBS (pH 7.4) and resuspended to obtain an OD_600 nm_ of 0.5. The ROS accumulation of *S. aureus* ATCC 29213 treated by AMG or IBC was measured with fluorescence probe DCFH‐DA (10 µmol L^−1^). Briefly, DCFH‐DA was added to the bacterial suspension and incubated at 37 °C for 20 min. After washing with 0.01 mol L^−1^ of PBS three times, 190 µL bacterial suspension was added to 96‐well plate and mixed with 10 µL AMG or IBC solution (final concentrations of 0.5, 1, 2, and 4 × MIC). After incubation for certain time, the fluorescence value was determined by Infinite M200 Microplate reader (Tecan), with the excitation wavelength at 488 nm and emission wavelength at 525 nm.

### Animal Studies

Female BALB/c and ICR mice aged 8–10 weeks and weighing ≈ 20 g from Beijing Vital River Laboratory Animal Technology Co., Ltd (Beijing, China) were used in this study. Mice were adapted to standardized environmental conditions (temperature = 23 °C ± 2 °C; humidity = 55% ± 10%) for one week before infection in China Agricultural University to minimize potential confounders. Mice were maintained in strict accordance with the regulations for the Administration of Affairs Concerning Experimental Animals approved by the State Council of People's Republic of China (11‐14‐1988). The animal study protocols were performed in accordance with the relevant guidelines and regulations (ID: SKLAB‐B‐2010‐003). The laboratory animal usage license number is SYXK‐2016‐0008, certified by Beijing Association for Science and Technology.

### Mouse Skin Wound Infection Model

BALB/c mice (*n* = 18 mice in each group) were anesthetized with pentobarbital (30 mg kg^−1^). The fur on the backs of the mice was removed by shaving, and 10 mm diameter wounds on the back were achieved using surgical punches. Then, 50 µL bacterial suspension containing 1.0 × 10^7^ CFUs MRSA T144 in PBS was inoculated onto each wound. At 1 h post‐infection, AMG (2 mg kg^−1^), IBC (8 mg kg^−1^), or vancomycin (1 mg kg^−1^) was administered to the wounds. Wound size was determined after treatments at the indicated times (1, 3, 6, 10, 13, and 15 days). And at relative days, wound tissues from three mice of each group were excised (including all accumulated pus), homogenized in sterile PBS, and the suspension was plated on *S. aureus* chromogenic agar plates (CHRO Magar, Paris, France) to count the bacterial burdens in the skin.

### VRE‐Colonized Model

ICR mice (*n* = 6 per group) were administered ampicillin (50 µg mL^−1^) in drinking water. After 5 days, the drinking water with ampicillin was replaced with sterile water. On day 6, mice went through intragastric administration with VRE_fm_CAU369 (1.0 × 10^9^ CFUs in 200 µL PBS). At 24 h post‐infection, mice went through intragastric administration with AMG (5 mg kg^−1^), IBC (20 mg kg^−1^), or tiamulin (5 mg kg^−1^). Fecal pellets were collected and resuspended in PBS to count the bacterial burdens before treatment (0 day) and after treatment of 7 days (1–7 days). Meanwhile, three mice in each group were sacrificed and the contents of ileum, cecum, and colon were collected to count the bacterial burdens of VRE_fm_ CAU369 at day 1 and day 7.

### Food Spoilage Model

To assess the potential of AMG or IBC as food preservatives, a pork spoilage model contaminated by MRSA T144 was established. Briefly, pork was divided into 12 parts (≈ 2.0 g each) and randomly divided into four groups (*n* = 4 per group). One group was with no treatment. The other three groups were loaded with 50 µL of MRSA T144 suspension (6 × 10^3^ CFUs per part) through infusion. Subsequently, 50 µL PBS, AMG (2 mg kg^−1^), or IBC (4 mg kg^−1^) was dropped. After culturing at 37 °C for 24 h, the meat was homogenized with sterile PBS and the suspensions were plated on MRSA chromogenic agar plates (CHRO Magar) to count the bacterial loads.

### Cooking Utensils Model

Overnight cultures of MRSA T144 were washed three times and resuspended with PBS. Lunch boxes (*n* = 3 per group) were sterilized with 70% ethanol, and then infected with 150 µL of 6.0 × 10^6^ CFUs mL^−1^ MRSA T144 at room temperature. After incubation for 30 min, each region was treated with 50 µL of AMG (2 µg mL^−1^), and IBC (8 µg mL^−1^). At the indicated time, the bacteria were collected, plated onto MHA plates, and the bacterial clones counted after incubation at 37 °C for 24 h.

### Statistical Analysis

The values reported were shown as mean ± standard deviation (s.d). Sample size (*n*) for each statistical analysis was 3 and the statistical significance was analyzed by the unpaired student's *t*‐test method and comparisons among more than two groups were obtained by one‐way ANOVA. *P* values were indicated in the figures. Not significant (*P* > 0.05) was indicated with n.s. Statistical significance in experimental data was determined using GraphPad Prism 7.0 software.

## Conflict of Interest

The authors declare no conflict of interest.

## Supporting information

Supporting Information

## Data Availability

The data that supports the findings of this study are available in the supplementary material of this article.
