# Supplementary material for: Plant Natural Flavonoids Against Multidrug Resistant Pathogens
Source: Adv Sci (Weinh). 2021 May 26;8(15):2100749. doi: 10.1002/advs.202100749 (PMC8336499; doi:10.1002/advs.202100749)
Supplement: Supplementary file 1 — Supporting Information [file ADVS-8-2100749-s001.pdf]

WILEY-VCH

© 2021 Wiley-VCH GmbH

ADVANCED SCIENCE
Open Access

Supporting Information

for *Adv. Sci.*, DOI: 10.1002/advs.202100749

Plant natural flavonoids against multidrug resistant pathogens

Meirong Song,[†] Ying Liu,[†] Tingting Li,[†] Xiaojia Liu, Zhihui Hao, Shuangyang Ding, Pharkphoom Panichayupakaranant, Kui Zhu,[*] Jianzhong Shen[*]

Supporting Information

Plant natural flavonoids against multidrug resistant pathogens

Meirong Song,[†] *Ying Liu*,[†] *Tingting Li*,[†] *Xiaojia Liu*, *Zhihui Hao*, *Shuangyang Ding*, *Pharkphoom Panichayupakaranant*, *Kui Zhu*,[*] *Jianzhong Shen*[*]

Dr M. R. Song, Y. Liu, Dr T. T. Li, X. J. Liu, Prof. K. Zhu, Prof. J. Z. Shen
National Center for Veterinary Drug Safety Evaluation, College of Veterinary Medicine, China Agricultural University, Beijing 100193, China
E-mail: zhuk@cau.edu.cn (K.Z.); sjz@cau.edu.cn (J.S.)

Prof. Z. H. Hao, Prof. K. Zhu
Center of Research and Innovation of Chinese Traditional Veterinary Medicine, China Agricultural University, Beijing 100193, China

Prof. S. Y. Ding, Prof. J. Z. Shen
Beijing Key Laboratory of Detection Technology for Animal-Derived Food Safety and Beijing Laboratory for Food Quality and Safety, China Agricultural University, Beijing 100193, China

Prof. P. Panichayupakaranant
Department of Pharmacognosy and Pharmaceutical Botany, Faculty of Pharmaceutical Sciences, Prince of Songkla University, Hat-Yai, Songkhla 90112, Thailand

†These authors contributed equally to this work.

Table S1. Bacteria strains used in this study.

Strains	Description	Reference
**Gram-positive bacteria**		
Staphylococcus aureus ATCC 29213	MSSA	[1]
S. aureus T144	MRSA [*mecA*, *tet(A)*]	[1]
S. aureus 65322	MRSA	This study
Enterococcus faecium CAU 382	VRE_{fm}	This study
E. faecium CAU 383	VRE_{fm}	This study
E. faecium CAU 369	VRE_{fm}	This study
**Gram-negative bacteria**		
Acinetobacter baumannii 17	*mcr-1*	[2]
Acinetobacter baumannii 7-2	Wildtype	This study
Acinetobacter baumannii 7-2 LPS-	LPS deficient; colistin resistant	This study
Citrobacter freumdii 2-4	*mcr-1*	[2]
Escherichia coli ATCC 25922		[2]
E. coli B2	*mcr-1*, *bla*$_{NDM-5}$, *bla*$_{TEM-1B}$, *bla*$_{OXA-10}$, *bla*$_{CTX-M-14}$, *tet(A)*, *aadA*, *ahp(4)*, *oqxAB*, *mdfA*, *fosA3*	[2]
E. coli G6	*bla*$_{NDM-5}$, *tet(A)*	[2]
E. coli 15-1x	Wildtype	This study
E. coli 6-2x	Wildtype	This study
E. coli ZJ134	*mcr-1*, *aac3*, *aadA2*, *aph(3)*, *ampC*, *bla*$_{CTX-M-14}$, *dfrA*, *fosA*, *mphA*, *strA*, , *tet(A)*, *flo*^{R}	[2-3]
E. coli ZJ33	*mcr-1*, *aac3*, *aadA2*, *aph(3)*, *aph(4)*, *ampC*, *bla*$_{CTX-M-64}$, *bla*$_{TEM-33}$, *mphA*, *oxa-10*, *fosA*, *tet(A)*	[2-3]
E. coli ZJ478	*mcr-1*, *aac3*, *aadA5*, *ampC*, *bla*$_{CTX-M-55}$,	[2-3]

	bla$_{ndm\text{-}5}$, *mphA, tet(A)*	
Enterobacter cloacae 16-15	*mcr-1*	[2]
Klebsiella pneumoniae ATCC 43816		[4]
K. pneumoniae 43816 Δ*waaC*	*waaC* knockout	This study
K. pneumoniae 4-20	*mcr-1*	[2]
Pseudomonas aeruginosa PAO1		[5]
Providencia alcalifaciens 16-1	*mcr-1*	[2]
Raoultella ornithinolytica 16-8	*mcr-1*	[2]
Raoultella planticola 16-15	*mcr-1*	[2]
*Serratia marcescens*16-99	*mcr-1*	[2]
Salmonella enteritidis SH30	*mcr-1*	[2]

ATCC, American Type Culture Collection; MRSA, methicillin-resistant *Staphylococcus aureus*; MSSA, methicillin-sensitive *Staphylococcus aureus*; VRE, vancomycin-resistant *Enterococcus faecium*; *mcr-1,* mobile colistin resistance gene; *tetA*, tetracyclines resistant gene; *bla*$_{NDM\text{-}5}$, carbapenems resistant gene; *bla*$_{TEM\text{-}1B}$, penicillins resistant gene; *bla*$_{OXA\text{-}10}$, carbapenems resistant gene; *bla*$_{CTX\text{-}M\text{-}14}$, *β*-lactams especially cefotaxim resistant gene; *aadA*, aminoglycosides resistant gene; *ahp(4)*, hygromycin B resistant gene; *oqxAB*, quinolones resistant gene; *mdfA*, cationic or zwitterionic lipophilic compounds resistant gene; *fosA3*, fosfomycin resistant gene.

Table S2. Antibacterial activities and prenylation characteristics of 85 flavonoids.

Compound	Prenylation		LogP[f]	MIC (μg mL^{-1})			Colistin[a]	FICI[b]	Plant origin
	n	Position		MSSA ATCC 29213	MRSA T144	*E. coli* B2			
**Flavones**									
Albanin A[c]	1	3	3.96	32	32	>128	4	0.53	*Artocarpus styracifolius, Artocarpus heterophyllus, Morus nigra, Artocarpus nitidus, Artocarpus gomezianus, Brosimopsis oblongifolia*[6-14]
Artocarpin[d]	2	3, 6	6.28	1	2	>128	0.25	0.06	*Artocarpus heterophyllus, Artocarpus gomezianus, Artocarpus nitidus*[6-8,10]
Broussoflavonol F[c]	2	3', 8	6.67	16	16	>128	4	0.53	*Macaranga indica, Macaranga barteri, Macaranga recurvata, Broussonetia papyrifera, Formosan Broussonrtia papyrifera, Tetragonula affbiroi*[15-20]
Corylifol C[c]	1	8	4.52	16	64	>128	8	1.03	*Psoralea corylifolia, Ershen pill*[21,22]
Isolicoflavonol[c]	1	3'	4.42	>128	>128	>128	8	1.03	*Glycyrrhiza uralensis, Daphne giraldii, Trollius chinensis, Scutellaria baicalensis, Macaranga indica, Engelhardtia roxburghiana, Glycyrrhiza glabra, Morus cathayana, Morus australis, Broussonetia papyrifera, Macatanga conifer, Platanus acerifolia*[19,23-29]
5'-Geranyl-5,7,2',4'-	2	5', 5'	6.27	2	2	>128	0.50	0.09	Morus lhou, Morus nigra[30,31]

Tetrahydroxy-flavone[c]									
Gancaonin O[c]	1	6	4.22	4	4	>128	2	0.28	Chemical synthesis
5,7,4'-Trihy-droxy-3,6-di-methoxy-3',5'-diprenylflavone[c]	2	3', 5'	6.29	64	32	>128	8	1.03	*Dodonaea viscosa, Dodonaea polyandra*[32,33]
8,3'-Diprenyl-apigenin[c]	2	3', 8	6.96	>128	>128	>128	4	0.53	*Epimedium koreanum, Epimedium agittatum, Morus alba, Epimedium btevicornum*[34-36]
3'-Geranyl-3-prenyl-5,7,2',4'-Tetrahydroxy-flavone[c]	3	3', 3', 3	7.54	4	4	>128	1	0.16	*Morus alba*[37]
Kuwanon A[d]	2	3', 3	5.84	4	8	>128	0.25	0.06	*Psoralea corylifolia, Morus atropurpurea, Morus australis, Morus lhou, Morus multicaulis, Morus alba*[38-41]
Licoflavone A[d]	1	6	5.00	>128	>128	>128	4	0.53	*Glycyrrhiza inflata, Glycyrrhiza uralensis*[42,43]
Licoflavone B[d]	2	3', 6	7.26	32	32	>128	4	0.53	*Glycyrrhiza inflata*[43-46]
Licoflavone C[d]	1	8	4.71	32	64	>128	8	1.03	*Morus alba, Retama raetam, Mori Cortex, Genista morisii, Genista ephedroides, Glycyrrhiza inflata*[43,45,47-51]
Licoflavonol[c]	1	6	4.42	8	8	>128	1	0.16	*Glycyrrhiza uralensis, Maclura tinctoria*[52-55]
Morin[d]	0	—	1.88	64	32	>128	16	2.03	*Cortex Mori*[54-57]
Morusin[d]	2	3, 8	5.64	8	8	>128	0.25	0.06	*Morus alba, Cortex Mori, Morus*

									atropurpurea, Mori Cortex[38,40,48,50,57-59]
Mulberrin[d]	2	3, 8	6.21	1	1	>128	0.25	0.06	*Morus alba, Morus atropurpurea*[57,60]
Oroxylin A[c]	0	—	2.96	>128	>128	>128	8	1.03	*Scutellaria baicalensis, Oroxylum indicum, Phyllodium pulchellum, Ligularia sagittal, Scutellaria luzonica, Scorzonera divaricata, Scutellaria intermedia, Lawsonia inermis, Astragalus membranaceus, Scutellariae radix, Scutellaria adenostegia, Kalimeris indica, Centaurea scoparia, Evodia lepta, Ornithogalum thyrsoides, Dichrocephala benthamii, Scutellaria regeliana, Centaurea omphalotricha, Scutellaria lateriflora, Lagochilus leiacanthus, Ardisia crispa, Eucommia ulmoides*[61-72]
6-Prenylapigenin[c]	1	6	4.71	32	32	>128	4	0.53	*Cudrania tricuspidata, Artocarpus heterophyllus, Garcinia xanthochymus, Dorstenia barteri, Dorstenia convexa, Dorstenia ciliata, Dorstenia dinklagei, Cannabis sativa*[6,73-79]
6-Prenyl-quercetin-3-methylether[c]	1	6	4.21	32	64	>128	8	1.03	*Hypericum japonicum, Glyyrrhiza uralensis, Sinopodophyllum emodi, Hypericum ightianum, Glycyrrhiza glabra*[53,80]
8-Prenyl-kaempferol[c]	1	8	4.42	32	32	>128	8	1.03	*Macaranga barteri, Sophora flavescens, Epimedium koreanum*[81-86]

5,7,2',4'-Tetra-hydroxy-3-geranylflavone[c]	2	3, 3	5.80	8	8	>128	8	1.03	*Broussonetia papyrifera*[87]
**Isoflavones**									
Eurycarpin A[c]	1	3'	4.52	8	8	>128	1	0.16	*Chemical synthesis*
Wighteone[c]	1	6	4.52	8	8	>128	2	0.28	*Pueraria peduncularis, Lotus japonicus, Erythrina stricta, Erythrina suberosa, Lupinus angustifolius, Pueraria mirifica, Glycyrrhiza uralensis, Maclura tinctoria, Cudrania tricuspidata, Ficus tikoua, Erythrina fusca, Erythrina poeppigiana, Psoralea corylifolia, Erythrina variegate, Cudrania fruticosa, Lipinus mutabilis, Bolusanthus speciosus, Cudrania cochinchinensis, Sicilian Glycyrrhiza, Erythrina lysistemon, Erythrina indica, Erythrina arborescens, Genista ephedroides, Anthyllis hermanniae, Erythrina orientalis, Laburnum anagyroids*[52,88-106]
Luteone[c]	1	6	4.23	64	64	>128	4	0.53	*Erythrina stricta, Lupinus angustifolius, Lupinus reflexus, Laburnum anagyroids*[89,99,101,107]
Sophora-isoflavone A[c]	1	3'	3.87	32	32	>128	4	0.53	*Sophora mooracrotiana*[108,109]
8-Prenyldaidzein[c]	1	8	4.81	32	32	>128	4	0.53	*Psoralea corylifolia, Erythrina fusca, Bituminaria morisiana, Bituminaria bituminosa, Erythrina indica,*

									Erythrina sigmoidea, Erythrina eriotricha[110-115]
Lupiwighteone[c]	1	8	4.52	32	>128	>128	8	1.03	*Vatairea guianensis, Glycyrrhiza glabra, Lotus pedunculatus, Ficus tikoua, Erythrina sigmoidea, Erythrina variegate, Glycyrrhiza glabra, Bolusanthus speciosus, Anthyllis hermanniae*[90,116-121]
Isowighteone[c]	1	3'	4.52	16	16	>128	4	0.53	*Lupinus albus, Psoralea corylifolia*[122]
6,8-Diprenyl-orobol[c]	2	6, 8	5.61	16	8	>128	8	1.03	*Cudrania tricuspidata, Flemingia philippinensis, Millettia pachycarpa, Glycyrrhiza uralensis, Maclura tinctoria*[104,123-126]
Isoneobava-isoflavone[c]	0		4.39	4	4	>128	0.5	0.09	*Psoralea corylifolia, Erythrina sigmoidea*[127,128]
8-Prenylluteone[c]	2	6, 8	5.80	8	8	>128	4	0.53	*Erythrina senegalensis, Erythrina burttii*[129]
Gancaonin M[c]	1	8	5.05	8	8	>128	8	1.03	*Deguelia costata*[130]
Corylin[d]	1	3'	4.24	>128	>128	>128	4	0.53	*Psoralea corylifolia, Psoraleae fructus, Eragrostis ferruginea, Erythrina sigmoidea*[131-135]
Isoflavone[c]	0		3.54	>128	>128	>128	8	1.03	*Ormosia henryi, Anaxagorea luzonensis, Dalbergia velutina, Peltophorum pterocarpum, Camphorosma lessingii, Pterocarpus marsupium, Milettia ferruginea, Pueraria lobata, Trifolium riograndense, Maclura tricuspidata, Ficus hispida*[136-142]

2,3-Dehydro-kievitone[c]	1	8	4.23	>128	>128	>128	4	0.53	*Maackia fauriei, Erythrina sacleuxii*[143,144]
Warangalone[c]	2	6, 8	5.73	8	2	>128	4	0.53	*Erythrina senegalensis, Cudrania tricuspidata, Maclura pomifera, Erythrina addisoniae, Erythrina sigmoidea, Millettia taiwaniana, Derris scandens*[145-151]
Lupalbigenin[c]	2	3', 6	6.77	1	1	>128	0.5	0.09	*Atalantia monophylla, Derris scandens, Cudrania tricuspidata, Ulex jussiaei, Sophora flavescens, Sophora microphylla, Anthyllis hermanniae*[104,152-158]
6,8-Diprenyl-genistein[c]	2	6, 8	6.09	4	2	>128	2	0.28	*Erythrina excelsa, Erythrina senegalensis, Cudrania tricuspidata, Streptococcus mutans, Glycyrrhiza uralensis, Erythrina caffra, Erythrina variegate, Erythrina sigmoidea, Erythrina eriotricha*[126,159-167]
6,7,4'-Tri-hydroxy-isoflavone[c]	0	—	2.07	64	16	>128	8	1.03	*Taxus media, Taxus mairei, Caragana changduensis, Bear bile, Soybean miso, Euchresta formosana, Fermented soybeans*[168-171]
Licoisoflavone A[d]	1	3'	4.23	32	32	>128	1	0.16	*Glycyrrhiza uralensis, Glycyrrhiza inflata, Azorella madreporica, Glycyrrhiza glabra, Lupinus texensis, Glycyrrhiza eurycarpa, Glycyrrhiza aspera, Echinosophora koreensis, Pueraria mirifica, Lupinus angustifolius, Phaseolus coccineus*[172-

									179]
Neobava-isoflavone[d]	1	3'	4.81	128	128	>128	16	2.06	*Psoralea corylifolia, Erythrina sigmoidea, Erythrina excels, Erythrina senegalensis, Psoraleae fructus, Hedan tablet, Lespedeza cyrtobotrya, Erythrina abyssinica, Erythrina latissimi, Sopubia delphinifolia*[180-182]
**Flavanones**									
Bavachin[d]	1	6	4.45	32	64	>128	1	0.16	*Psoralea corylifolia, Psoralea fructus, Millettia speciosa, Piper longum*[183]
Bavachinin A[d]	1	6	4.52	4	8	>128	1	0.16	*Psoralea corylifolia, Psoralea fructus*[184]
Glabrol[d]	2	3', 8	6.70	1	1	>128	0.12	0.06	*Glycyrrhiza uralensis, Vernonia cinerea, Glycyrrhiza glabra, Glycyrrhiza inflata, Sophora tonkinensis, Astragalus monbeigii, Pistacia lentiscus, Pseudosophora alopecuroides, Sophora tonkinensis, Erythrina subumbrans, Euchresta formosana, Sophora prostrate, Euchresta japonica, Euchresta horsfieldii, Glycyrrhiza eurycarpa*[185-192]
Isobavachin[d]	1	8	4.45	128	128	>128	1	0.16	*Psoralea corylifolia, Psoralea fructus, Sanicula lamelligera, Erythrina sigmoidea*[53,117,191,193]
Liquiritigenin[d]	0	—	2.20	>128	>128	>128	4	0.53	*Erinacea anthyllis, Dalbergia tonkinensis, Erythrina caffra, Curcuma rotunda, Praxelis clematidea, Physails*

									alkekengi, Gansuibanxia decoction, Spatholobus suberectus, Milettia dielsiana, Areca catechu, Millettia speciosa, Glycyrrhiza uralensis, Vigna luteola, Bauhinia forficata, Endocomia macrocoma, Licorice residues, Pongamia pinnata[53,194-203]
6-Prenyl-naringenin**[d]**	1	6	4.16	8	8	>128	0.5	0.09	*Glycyrrhiza uralensis, Mallotus conspurcatus, Cudrania tricuspidata, Humulus lupulus, Glycyrrhiza inflata, Aspergillus fumigatus, Psoralea corylifolia* [131,204-209]
Cudra-flavanone B**[c]**	1	6	4.08	>128	>128	>128	8	1.03	*Cudrania tricuspidata, Cudrania cochinchinensis*[210]
3,7,4'-Tri-hydroxy-5-methoxy-8-prenylflavanone**[c]**	1	8	3.52	>128	>128	>128	8	1.03	*Sophora flavescens*[211]
Euchresta-flavanone A**[c]**	2	3', 8	6.62	2	2	>128	2	0.28	*Azadirachta indica, Euchresta formosana, Sophora species, Macaranga pleiostemona, yellow lupin*[78,212-215]
Sophora-flavanone C**[c]**	2	8, 8	6.13	2	2	>128	1	0.16	*Drynaria fortune, Sophora tomentosa, Echinosophora koreensis*[173,216,217]
Leachianone G**[c]**	1	8	4.28	32	32	>128	4	0.53	*Podocarpium podocarpum, Desmodium caudatum, Desmodium caudatum, Cudrania tricuspidata, Sophora flavescens, Lespedeza*

									cyrtobotrya, Morua alba, Sophora leachiana[218-222]
Licoflavanone[c]	1	3'	4.71	32	32	>128	4	0.53	*Glycyrrhiza glabral*[223]
Sophoranone[c]	3	3', 5', 8	8.24	>128	>128	>128	4	0.53	*Sophora tonkinensis, Sophara subprostrata*[187,224,225]
**Chalcone**									
Isobavachalcone[d]	1	5'	4.81	4	4	>128	0.06	0.04	*Dorstenia barteri* [73,76,226-228]
Xanthohumol[d]	1	5'	4.80	4	4	>128	0.12	0.06	*Sophora flavescens, Hunulus lupulus*[229]
4-Hydroxy-derricin[c]	1	5'	4.88	2	2	>128	0.5	0.09	*Angelica keiskei, Bifidobacterium dolescentis*[230,231]
Desmethyl-xantholhumol[c]	1	3'	4.73	16	16	>128	2	0.28	*Humulus lupulus*[232-235]
Kanzonol C[c]	2	5, 5'	7.06	4	4	>128	0.25	0.06	*Glycyrrhiza inflata, Dorstenia barteri, Dorstenia turbinata, Glycyrrhiza eurycarpa*[43,236,237]
Licochalcone A[d]	1	3	4.85	4	4	>128	0.5	0.09	*Glycyrrhiza glabra* [53]
Licochalcone C[d]	1	5	4.91	4	4	>128	0.5	0.09	*Glycyrrhiza uralensis, Glycyrrhiza radix, Glycyrrhiza inflata, Glycyrrhiza glabra*[178,238,239]
Licochalcone D[d]	1	3'	4.65	16	32	>128	8	1.03	*Glycyrrhiza inflata*[240]
Licochalcone E[d]	1	3	4.75	4	4	>128	0.12	0.06	*Glycyrrhiza inflata*[241]
Xanthoangelol[c]	2	5', 5'	6.66	32	>128	128	＞8	2.03	*Angelica keiskei, Amorpha fruticosa, Psoralea corylifolia, Lespedeza cyrtobotrya, Artocarpus nobilis*[227,242,243]
**Xanthones**									
α-Mangostin[d]	2	2, 8	6.32	1	1	>128	0.06	0.04	*Cratoxylum cochinchinense, Garcinia travancorica, Garcinia mangostana,*

									Calphyllum tetrapterum, Garcinia tetrandra, Mangifera indica, Inula viscosa, Garcinia cowa, Garcinia fusca, Garcinia iowar, Garcinia oblongifolia, Tetranula laeviceps, Garcinia pedunculata, Comastoma pedunculatum, Cratoxylum arborescens, Garcinia hombroniana, Calophyllum brasiliense, Mesua ferrea, Mesua congestiflora[244-260]
γ-Mangostin[c]	1	2	6.05	2	1	>128	8	1.03	*Garcinia travancorica, Garcinia angostana, Hypericum perforatum, Garcinia esculenta, Garcinia cowa, Cratoxylum cochinchinense, Garcinia xipshuanbannaensis, Garcinia costata, Cratoxylum formosum, Hypericum androsaemum*[253,261-268]
1,3,7-Trihydroxy-2-prenylxanthone[c]	1	2	4.52	16	16	>128	2	0.28	*Cudrania cochinchinensis*[266]
1,4,5,6-Tetra-hydroxy-7,8-diprenyl-xanthone[c]	2	7, 8	6.07	>128	>128	>128	8	1.03	*Garcinia xanthochymus*[269]
1,4,5,6-Tetra-hydroxy-7-prenyl-xanthone[c]	1	7	4.29	128	16	>128	8	1.03	*Garcinia bracteata, Garcinia xanthochymus*[269-271]
1,4,6-Trihydroxy-5-methoxy-7-prenylxanthone[c]	1	7	4.56	128	128	>128	8	1.03	*Garcinia xanthochymus, Morus tropurpurea, Comastoma pulmonarium, Garcinia oblongifolia,*

									Garcinia bracteata, Garcinia dulcis[270,272-276]
8-Deoxygartanin[c]	2	2, 4	6.33	>128	>128	>128	8	1.03	*Garcinia mangostin, Endodesmia calophylloides, Garcinia merguensis*[271,277-281]
9-Hydroxy-calabaxanthone[c]	2	2, 8	5.75	8	2	>128	4	0.53	*Garcinia mangostin, Garcinia multiflora, Garcinia cowa*[282-288]
Garcinia-xanthone E[c]	3	7, 7, 8	7.65	>128	>128	>128	8	1.03	*Garcinia subelliptica*[289]
Gartanin[c]	2	2, 4	6.07	>128	128	>128	8	1.03	*Garcinia mangostana, Garcinia bracteata, Garcinia propinqua, Garcinia achachairu, Tetragonula laeviceps, Tetrigona melanoleuca, Garcinia nobilis, Garcinia staudtii, Garcinia dulcis, Maclura tinctoria*[157,290-297]
Isogarcinia-xanthone E[c]	3	4, 7, 8	8.06	16	2	>128	8	1.03	*Calophyllum elatum*[294]
Nujiangxanthone I[e]	3	4, 7, 8	6.71	64	16	>128	8	1.03	*Garcinia nujiangensis*[298]
Nujiangxanthone D[e]	2	4, 8	5.82	1	2	>128	8	1.03	*Garcinia nujiangensis*[298]
Isojacareubin[e]	1	4	3.69	16	16	>128	8	1.03	*Garcinia nujiangensis, Hypericum stellatum, Garcinia fusca, Garcinia livingstonei, Hypericum japonicum, Hyperium roeperanum, Garcinia schomburgkiana, Garcinia cowa, Garcinia xipshuanbannaensis*[80,298-303]
Nujiangxanthone	3	4, 7, 8	6.98	4	8	>128	8	1.03	*Garcinia nujiangensis*[298]

N[e]									
Buchana-xanthone[e]	0	—	2.81	8	16	>128	8	1.03	*Garcinia nujiangensis, Garcinia tetralata*[298,304]
6-Deoxy-isojacareubin[e]	1	4	4.19	>128	>128	>128	8	1.03	*Garcinia nujiangensis, Clusia burlemarxii, Garcinia nervosa, Hypericum laricifolium, Garcinia livingstonei, Allanblackia gabonensis, Vismia laurentii, Garcinia merguensis, Hypericum japonicum, Cudrania tricuspidata, Anaxagorea luzonensis, Cudrania tricuspidata, Allanblackia floribunda, Garcinia bracteate, Garcinia speciose, Garcinia celebica, Arenaria serpyllifolia, Garcinia amplexicaulis, Calophyllum thorelii, Garcinia xanthochymus, Vismia guineensis, Garcinia nigrolineata, Hypericum wightianum*[281,298,305-312]
Nujiangxanthone A[e]	3	4, 7, 8	7.84	2	8	>128	8	1.03	*Garcinia nujiangensis*[298]
Nujiangxanthone B[e]	3	4, 7, 8	8.11	64	16	>128	8	1.03	*Garcinia nujiangensis*[298]

[a] MIC (μg mL^{-1}) of colistin against *E. coli* B2 under 8 μg mL^{-1} flavonoids. [b] FICI of colistin and flavonoids (8 μg mL^{-1}) against *E. coli* B2 were shown. [c] Compounds were purchased from Shanghai Universal Biotech Company with a purity of ≥ 95% (Shanghai, China). [d] Compounds were purchased from Chengdu Biopurify Phytochemicals Ltd. with a purity of ≥ 95% (Chengdu, China). [e] Compounds were donated by Professor Hua Huiming of Shenyang Pharmaceutical University (Shenyang, China), Which were isolated from the twigs and leaves of *Garcinia nujiangensis* with a purity of ≥ 95%[293]. [f] The values of LogP were calculated using software Molinspiration.

Table S3. SAR-based molecular signatures of prenylated flavonoids and antimicrobial potency against Gram-positive bacteria.

Lead compound	Prenylation		MIC (μg mL^{-1})		
	n	Position	MSSA ATCC 29213	MRSA T144	VRE$_{fm}$ CAU 369
Mulberrin	2	3, 8	1	1	2
Lupalbigenin	2	6, 3 '	1	1	2
Glabrol	2	8, 3 '	1	1	2
Isobavachalcone	1	5 '	4	4	1
α-Mangostin	2	2, 8	1	1	2

Table S4. Synergy between lead compounds and colistin against Gram-negative bacteria.

Selected compound	Prenylation		MIC (μg mL^{-1})					
	n	position	*K. pneumoniae* ATCC 43816	*A. baumannii* 7-2	*P. aeruginosa* PAO1	*E. coli* B2	Flavonoids[a]	FICI[b]
Mulberrin	2	3, 8	>128	>128	>128	>128	0.25	0.06
Lupalbigenin	2	6, 3 '	>128	>128	>128	>128	0.50	0.09
Glabrol	2	8, 3 '	>128	>128	>128	>128	0.12	0.05
Isobavachalcone	1	5 '	>128	>128	>128	>128	0.06	0.04
α-Mangostin	2	2, 8	>128	>128	>128	>128	0.06	0.04

[a] The MIC of colistin under 4 μg mL^{-1} of flavonoids against *E. coli* B2.

[b] The FICI of flavonoids and colistin under 4 μg mL^{-1} of flavonoids against *E. coli* B2.

Table S5. Antibacterial activities of AMG/IBC against 23 clinical MRSA isolates.

Clinical MRSA isolates	MIC (μg/mL)	
	AMG	IBC
Human Origin (n = 7)		
03044	0.5	4
65322	0.5	4
65339	0.5	4
65543	0.5	4
6559	0.5	4
70675	0.5	1
70678	0.5	4
Animal Origin (n = 16)		
1518	0.5	4
1530	0.5	4
AB18	0.5	4
EF-2	0.5	4
HN3	0.5	4
L26	0.5	4
M7	0.5	8
NB61	0.5	4
NX-75	0.5	4
SB14	0.5	8
SB-27	0.5	4
T144	0.5	4
T182	0.5	4
T50	0.5	4
T52	0.5	4
ZS7	0.5	4
MIC_{50}	0.5	4
MIC_{90}	0.5	4

Table S6. Antibacterial activities of AMG/IBC against 50 clinical VRE isolates.

Clinical VRE_{fm} isolates	MIC (μg mL^{-1})		
	AMG	IBC	Vancomycin
CAU379	0.5	8	> 128
CAU380	0.5	4	> 128
CAU381	0.5	8	> 128
CAU382	0.5	8	> 128
CAU383	0.5	4	> 128
CAU384	0.5	8	> 128
CAU385	0.5	2	> 128
CAU386	0.5	4	> 128
CAU387	0.5	2	> 128
CAU388	0.5	8	> 128
CAU389	0.5	4	> 128
CAU393	0.5	8	> 128
CAU394	0.5	2	> 128
CAU399	0.5	2	> 128
CAU401	1	2	> 128
CAU402	1	2	> 128
CAU403	1	8	> 128
CAU404	1	4	> 128
CAU405	0.25	4	> 128
CAU406	0.5	8	> 128
CAU407	0.5	4	> 128
CAU408	0.25	4	> 128
CAU409	0.25	8	> 128
CAU410	0.5	4	> 128
CAU411	0.25	8	> 128
CAU412	0.5	8	> 128
CAU414	0.5	8	> 128

continued

Table S6 continued

CAU415	0.5	8	> 128
CAU416	0.5	8	> 128
CAU417	0.5	8	> 128
CAU421	0.5	4	> 128
CAU422	0.5	4	> 128
CAU424	0.5	8	> 128
CAU431	0.5	8	> 128

CAU433	0.5	8	> 128
CAU434	0.25	4	> 128
CAU436	0.5	8	> 128
CAU438	0.5	8	> 128
CAU442	0.5	4	> 128
CAU443	0.5	8	> 128
CAU445	0.5	4	> 128
CAU446	0.5	8	> 128
CAU447	0.5	8	> 128
CAU450	0.5	8	> 128
CAU454	0.5	8	> 128
CAU455	0.5	8	> 128
CAU456	0.5	8	> 128
CAU453	0.5	8	> 128
CAU457	0.5	8	> 128
CAU458	0.5	8	> 128
MIC_{50}	0.5	8	> 128
MIC_{90}	0.5	8	> 128

Table S7. The MIC of AMG/IBC against different Gram-negative bacteria.

Strains	Description	MIC (μg mL^{-1})		
		AMG	IBC	Colistin
K. pneumoniae ATCC 43816	Wild type	>128	>128	16
K. pneumoniae ATCC 43816 Δ*waaC*	*waaC* deficient	4	4	0.25
A. baumannii 7-2	Wild type	>128	>128	0.0625
A. baumannii 7-2 LPS-	LPS deficient	0.125	0.125	＞32

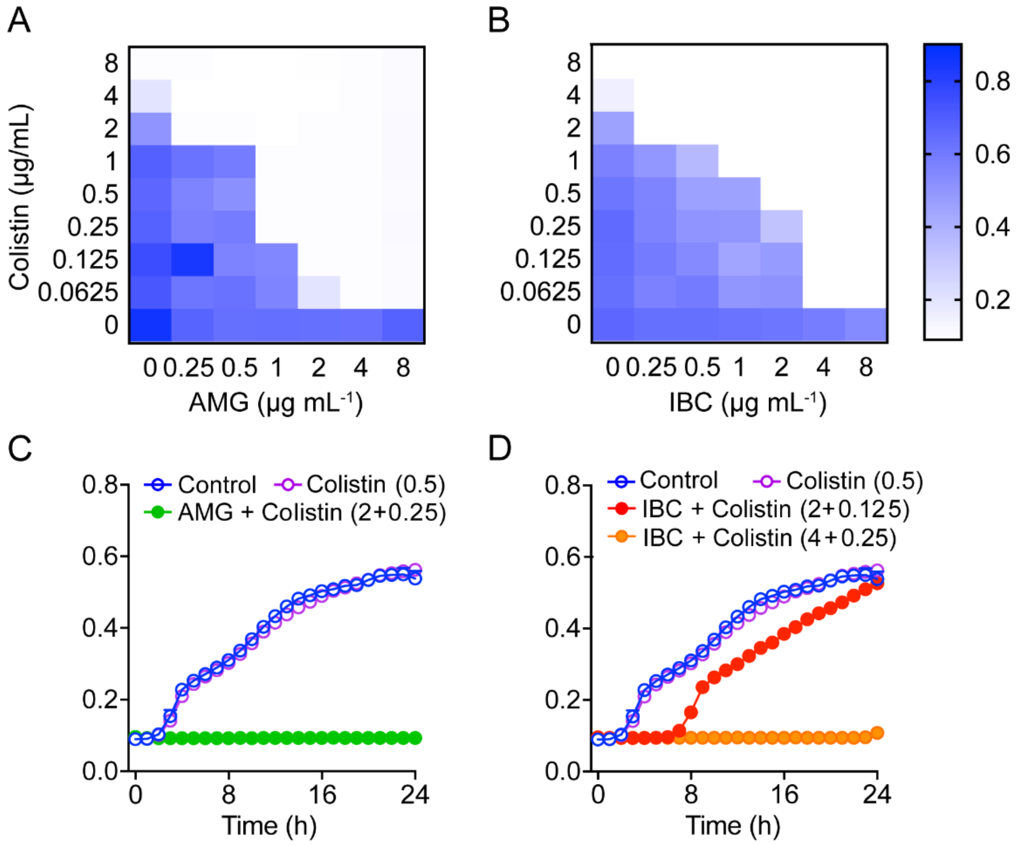

Figure S1. Combinations of AMG/IBC with colistin are effective against *E. coli* B2. A, B) Checkerboard microdilution assays showed the dose-dependent potentiation of colistin by AMG (A) or IBC (B) against *mcr-1* positive *E. coli* B2. Dark-blue regions represent higher cell density and lower inhibition rate of combinational treatment. Data represent two biological replicates, n = 3.

C, D) Kinetic analysis of AMG (C) or IBC (D) and colistin at different concentrations in *mcr-1* positive *E. coli* B2. Optical density (OD) at 600 nm was monitored once every hour for 24 h. Experiments were performed as three biologically independent experiments, and the mean ± s.d. is shown, n = 3.

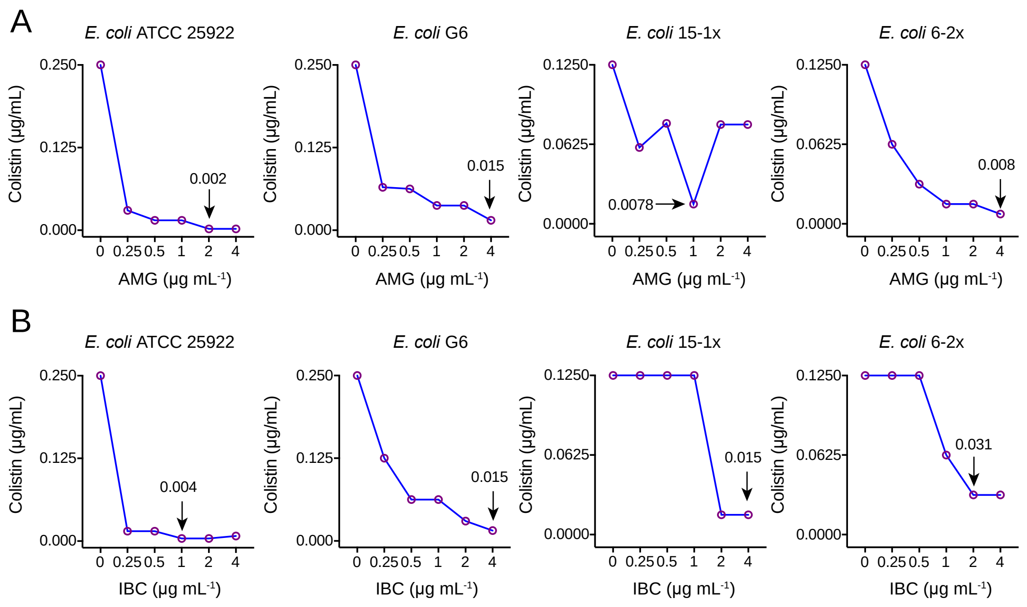

Figure S2. The synergy of AMG/IBC with colistin against colistin-sensitive *E. coli* isolates.

Sub-MIC levels of AMG (A) or IBC (B) enhanced the activity of colistin against four colistin-sensitive (*E. coli* ATCC25922, *E. coli* G6, *E. coli* 15-1x and *E. coli* 6-2x) by chequerboard microdilution assay. Experiments were performed as three biologically independent experiments, n = 3.

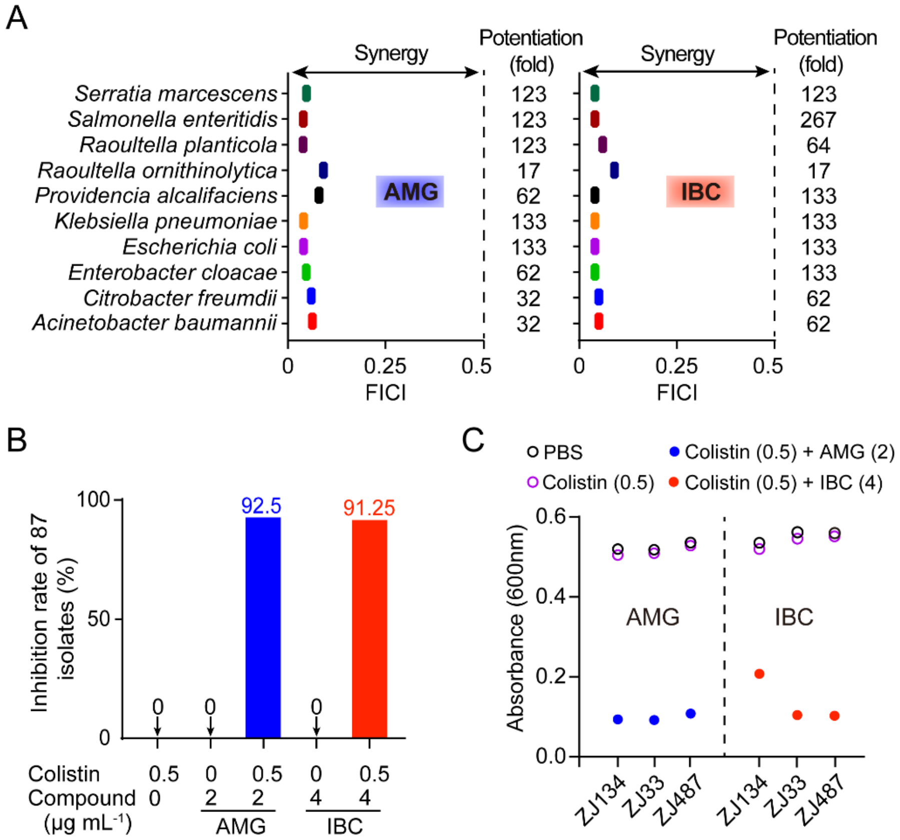

Figure S3. AMG or IBC restored the activity of colistin against colistin-resistant Gram-negative bacteria.

A) FICI values of colistin and AMG or IBC against 10 species of Gram-negative bacteria harboring *mcr-1*. The potentiation (fold) referred to the change in MIC of colistin under 8 μg mL^{-1} flavonoids against different isolates. Data represent two biological replicates, n = 3.

B) Combinations of AMG (2 μg mL^{-1}) or IBC (4 μg mL^{-1}) with colistin (0.5 μg mL^{-1}) against 87 *E. coli* isolates carrying *mcr-1*.[2-3] Growth inhibition was defined as no visible growth of bacteria treated with the combinations after incubation at 37 °C for 18 h. At least 72 of the 87 *mcr-1 E. coli* isolates are resistant to multiple classes of antibacterial agents except colistin. [3]

C) Combinations of AMG (2 μg mL^{-1}) or IBC (4 μg mL^{-1}) with colistin (0.5 μg mL^{-1}) against 3 multi-drug resistant *E. coli* isolates carrying *mcr-1*. $OD_{600\ nm}$ of bacteria sus suspension treated with the combinations after incubation at 37 °C for 18 h were determined. PBS was used as negative control. *E. coli* ZJ134, *E. coli* ZJ33 and *E. coli* ZJ487 carry 29, 31 and 20 antibiotic resistant genes, respectively. [3]

Experiments were performed as three biologically independent experiments, n = 3.

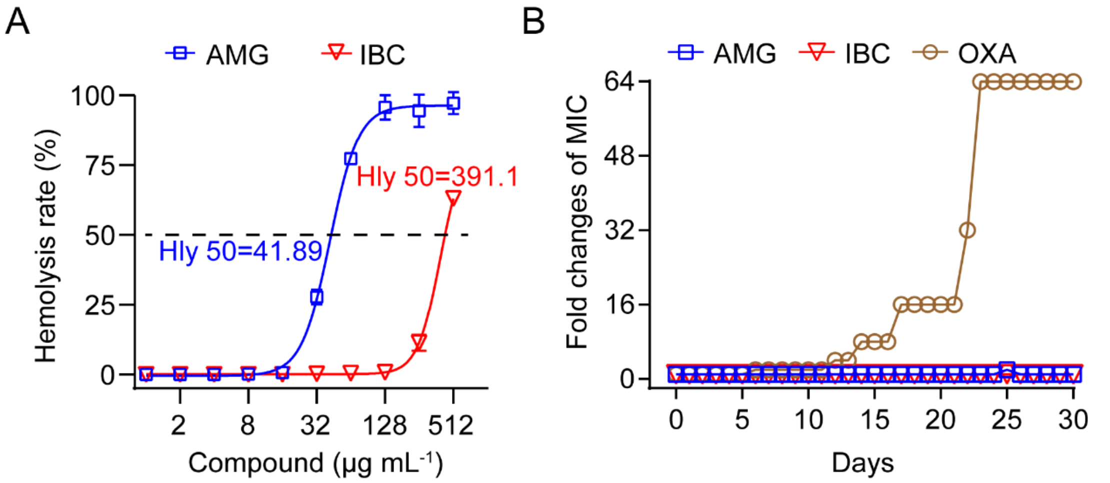

Figure S4. Safety evaluation and resistance development of AMG/IBC.

A) Hemolytic activity of AMG or IBC at different concentrations (0 - 512 μg mL^{-1}) to the red blood cells of sheep. AMG had low hemolysis (25.86%) at 32 μg mL^{-1}, while IBC showed no hemolysis (0.73%) even at high concentrations of 128 μg mL^{-1}. Experiments were performed as three biologically independent experiments. Data presented as mean ± s.d, n = 3.

B) No resistance to AMG or IBC occurred in 30-day serial passage. *S. aureus* ATCC 29213 was passaged daily in sub-inhibitory concentrations of AMG, IBC or oxacillin (OXA). OXA was used as positive control.

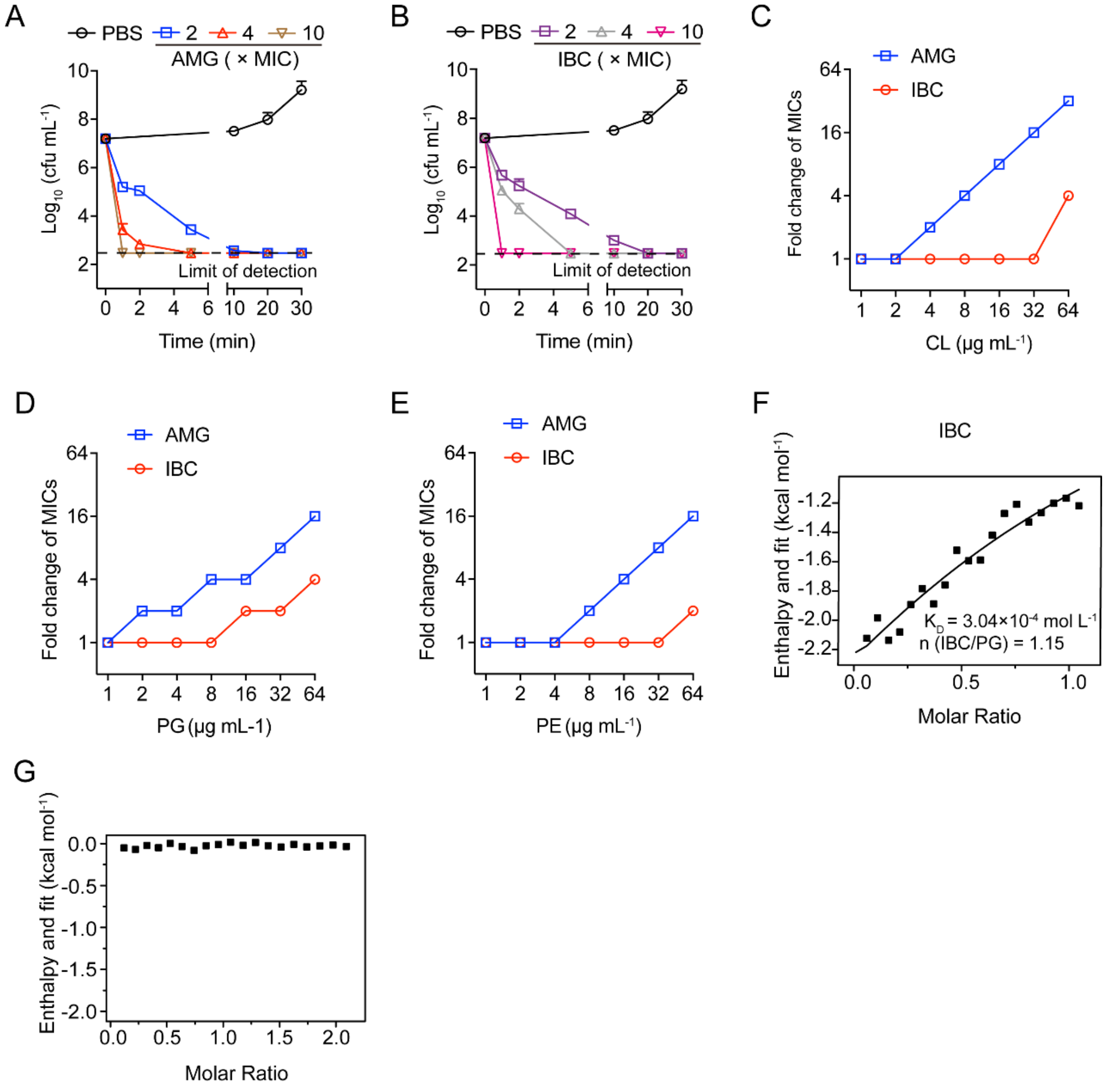

Figure S5. Antibacterial activities of AMG/BC through targeting phospholipids in bacterial membrane.

A, B) Time-killing curves of AMG or IBC against *S. aureus* ATCC 29213 at exponential phase at 37 °C. *S. aureus* cells were treated with 2, 4 and 10 × MIC of AMG (A), IBC (B) for 30 min, and the number of viable cells were determined at different time points.

C, D, E) Exogenous addition of CL (C), PG (D) and PE (E) decreased the antibacterial activity of AMG or IBC against *S. aureus* ATCC 29213 by chequerboard microdilution assays. The concentrations of lipids was in the range of 0 to 128 μg mL^{-1}. CL, PE and PG represent cardiolipin, phosphatidylethanolamine and phosphatidylglycerol, respectively.

F) ITC analysis of the interaction between PG and IBC. Thermodynamic parameters were calculated, including equilibrium dissociation constant (K_D = 3.04× 10^{-4} mol L^{-1}), number of binding sites (n = 1.15), molar binding enthalpy (ΔH = -21.57 kJ mol^{-1}) and molar binding entropy (ΔS = 5.87 J mol^{-1} K^{-1}).

G) ITC analysis of the interaction between PG and buffer (20 mmol L^{-1} HEPES, pH 7.0).

Experiments in A and B were performed as three biologically independent experiments, data represent as mean ± s.d, n = 3. Experiments in C-G were performed as two biologically independent experiments.

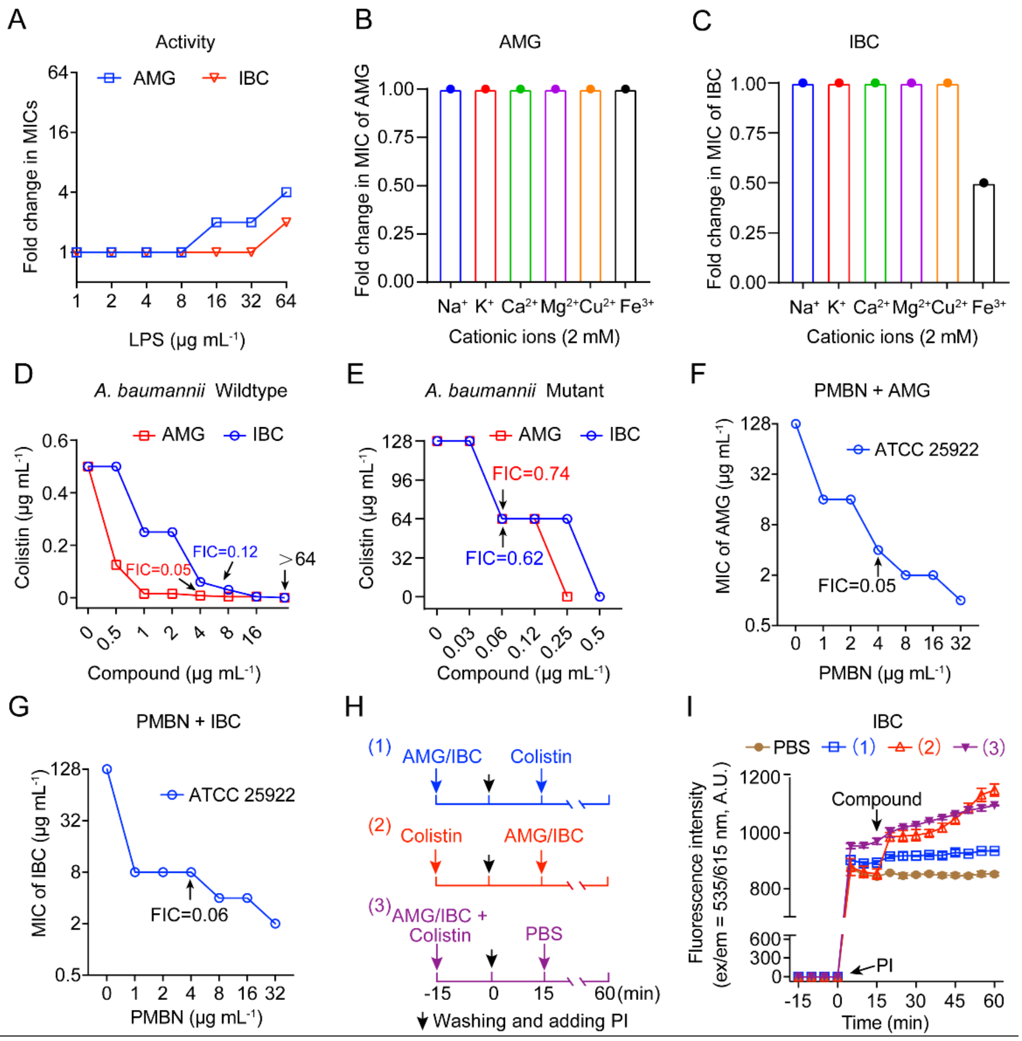

Figure S6. AMG/IBC exerts antibacterial effects through targeting membranes in Gram-negative bacteria.

A) The change in MIC of AMG or IBC against *S. aureus* ATCC 29213 in the presence of lipopolysaccharide (LPS) of the outer membrane. High levels of LPS had neglectable influence on the activity of either AMG or IBC.

B, C) Effects of divalent cations on the MICs of AMG (B) and IBC (C). Divalent cations had neglectable influence on the antibacterial activity of either AMG or IBC against *S. aureus* ATCC 29213.

D, E) Synergy of AMG and IBC with colistin for *A. baumannii* wildtype (D) and mutant (LPS-deficient) isolates (E). No synergy was observed after the LPS was deficient.

F, G) Synergy of the colistin analogue, polymyxin B nonapeptide (PMBN), with AMG (F) or IBC (G) against *E. coli* ATCC 25922 by checkerboard microdilution

assay.

H) The schedule of experimental design. *E. coli* B2 was incubated with different antibacterial drugs at 37 °C for 15 min. After washing for three times, PI was added and the fluorescence was determined for 15 min. Then the other drugs were added. The fluorescence was measured with the excitation wavelength at 535 nm and emission wavelength at 615 nm.

I) The permeability dynamics of inner membrane in *E. coli* B2, under the treatments of IBC, and colistin and both thereof. The concentrations of IBC and colistin were 4 and 0.25 μg mL^{-1}, respectively.

Experiments in A-G were performed as two biologically independent experiments, n = 3. Experiments in I were performed as three biologically independent experiments, data presented as mean ± s.d, n = 3.

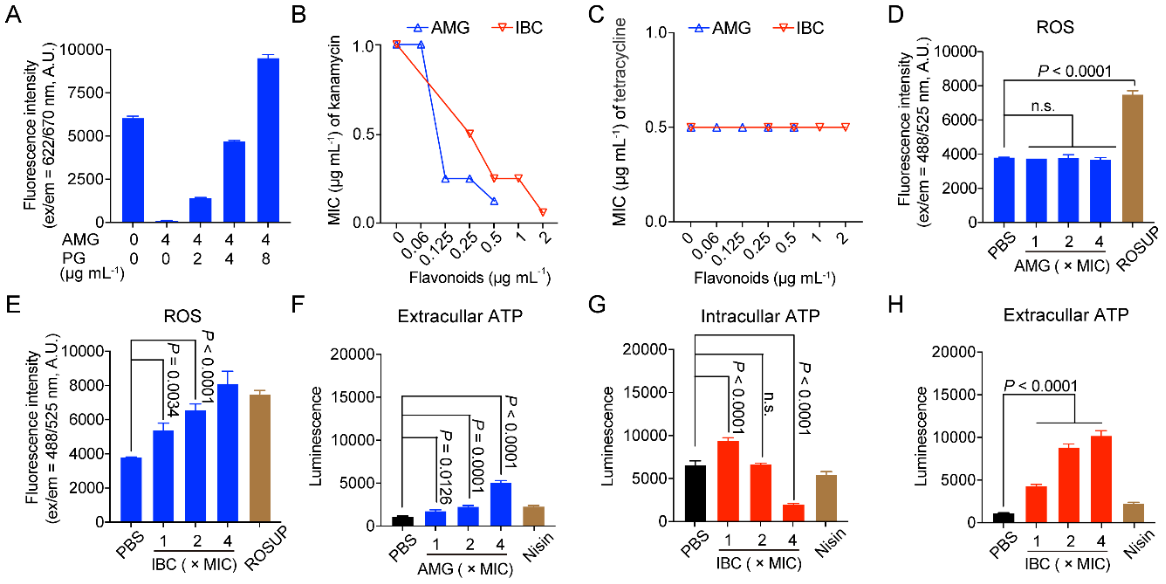

Figure S7. Mechanism of AMG/IBC against Gram-positive bacteria.

A) Competition kinetics between $DiSC_3$ (5) (1 μmol mL^{-1}), POPG (0-8 μg mL^{-1}) and AMG (4 μg mL^{-1}). The fluorescence of $DiSC_3$ (5) was determined by Infinite M200 Microplate reader (Tecan), with excitation wavelength at 622 nm and emission wavelength at 670 nm.

B) The synergy of kanamycin in combination with either AMG or IBC against *S. aureus* ATCC 29213.

C) No synergy was observed between AMG/IBC and tetracycline against *S. aureus* ATCC 29213.

D-E) The effect of AMG (C) or IBC (D) on the ROS accumulation in *S. aureus* ATCC 29213 after treatment for 15 min. ROSUP was used as the positive control.

F) Increased levels of extracellular ATP in *S. aureus* ATCC 29213 after the treatment of AMG for 10 min.

G, H) Decrease levels of intracellular (G) and increased levels of extracellular (H) ATP in *S. aureus* ATCC 29213 after the treatment of IBC for 10 min.

Experiments in A, D-H were performed as three biologically independent experiments, data presented as mean ± s.d, n = 3. *P*-values in C-G were calculated using non-parametric one-way ANOVA. Experiments in B-C were performed as three biologically independent experiments, n = 3.

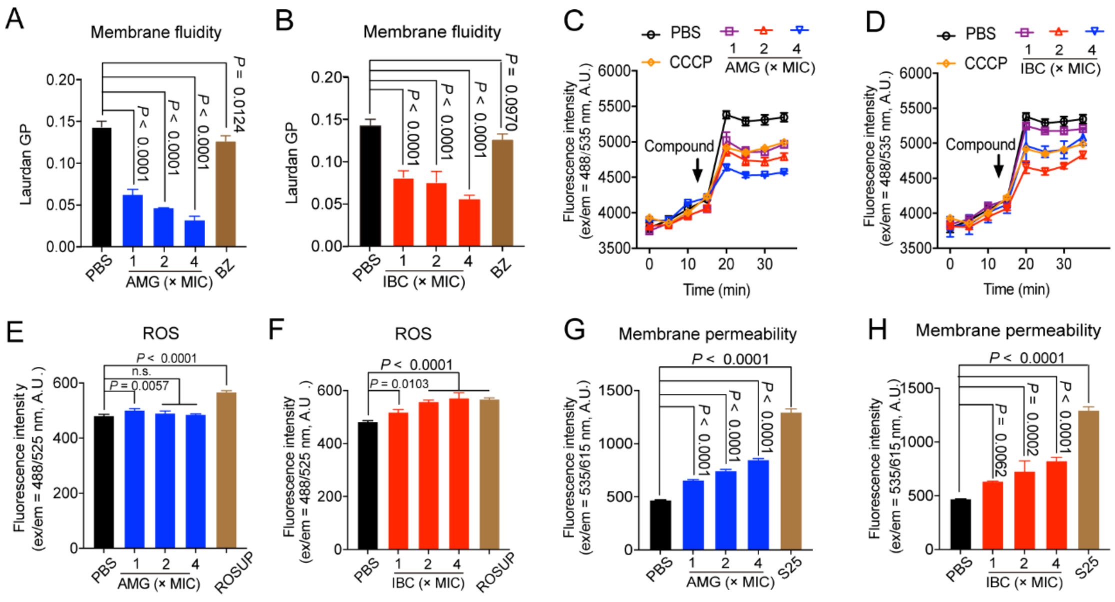

Figure S8. Mechanism of AMG or IBC against Gram-negative bacteria.

A, B) The membrane fluidity for *K. pneumonia* ATCC 43816 Δ*waaC* after treatment of AMG (A) or IBC (B) was increased. 50 mmol L^{-1} benzyl alcohol (BA) was used as the positive control.

C, D) Dissipated ΔpH by AMG (C) or IBC (D) in *K. pneumonia* ATCC 43816 Δ*waaC*. Exponential *K. pneumonia* ATCC 43816 Δ*waaC* was incubated with pH fluorescence probe BCECF-AM. After washed for three times, different concentrations of AMG or IBC were added and the intracellular pH was determined by measuring the fluorescence intensity with the excitation/emission wavelength at 488 nm/535 nm.

E, F) The effect of AMG (E) or IBC (F) on the ROS accumulation in *K. pneumonia* ATCC 43816 Δ*waaC*. ROSUP was used as the positive control.

G, H) Increased permeability of inner membrane for *K. pneumonia* ATCC 43816 Δ*waaC* after treatment of AMG (G) or IBC (H) for 20 min. SLAP-S25 was used as the positive control.

Experiments in A-H were performed as three biologically independent experiments, data presented as mean ± s.d, n = 3. *P*-values were calculated using non-parametric one-way ANOVA.

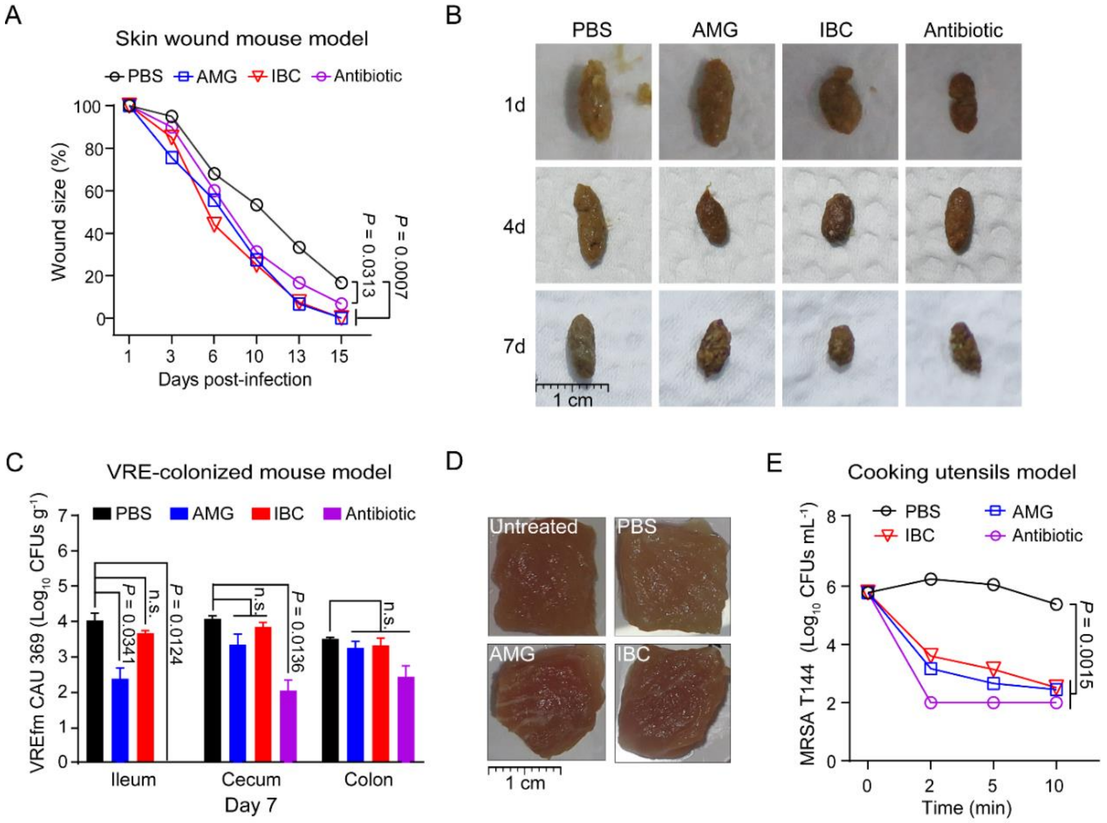

Figure S9. AMG and IBC exerted greatly antibacterial potential in four models.

A) Mouse skin wound infection models (n = 18 in each group). Skin wounds size reduced under the treatment of AMG (2 mg kg^{-1}) and IBC (8 mg kg^{-1}) on days 1, 3, 6, 10, 13 and 15 after injury in MRSA T144 (1.0×10^7 CFUs infection).

B) VRE-colonized mouse model. Representative photographs of fecal pellets on days 1, 4 and 7 under the treatment of AMG (5 mg kg^{-1}) and IBC (20 mg kg^{-1}). AMG and IBC reduced the symptoms of diarrhea in mice. Antibiotic (tiamulin, 5 mg kg^{-1}) was used as a positive control.

C) VRE-colonized mouse model. AMG and IBC decreased the numbers of VRE_{fm} CAU369 in ileum, cecum, and colon after treatment for 7 days. Antibiotic (tiamulin, 5 mg kg^{-1}) was used as a positive control.

D) Food spoilage model. Representative photographs of morphological characteristics of meat under different treatments at 37 °C for 24 h. The concentrations of AMG and IBC were 2 mg kg^{-1} and 4 mg kg^{-1}, respectively.

E) Cooking utensils model. Plastic lunch boxes were contaminated with MRSA T144 (6.0×10^6 CFUs) for 30 min. After treatment of AMG (2 μg mL^{-1}) or IBC (8 μg mL^{-1})

for 0, 2, 5, and 10 min, the bacteria number on the lunch boxes was calculated by the chromogenic agar plate.

Data presented as mean ± s.d, n = 3. *P*-values were calculated using non-parametric one-way ANOVA.

References

[1] Y. Liu, S. Ding, R. Dietrich, E. Martlbauer, K. Zhu, Angew. *Chem. Int. Ed. Engl.* **2017**, *56*, 1486.

[2] M. R. Song, Y. Liu, X. Y. Huang, S. Y. Ding, Y. Wang, J. Z. Shen, K. Zhu, *Nat. Microbiol.* **2020**, *5*, 1040.

[3] Y. B. Shen, Z. W. Wu, W. Yang, Z Rong, H. W. Zhou, S. L. Wang, L. Lei, M. Li, J. C. Cai, J. Tyrrell, G. B. Tian, C. M. Wu, Q. J. Zhang, J. Z. Shen, T. R. Walsh, Z. Q. Shen, *mBio.* **2018**, *9*, e00943-18.

[4] Z. Q. Yang, Q. L. Sun, S. Chen, S. Y. Ding, R. Zhang, K. Zhu, *Microb. Drug. Resist.* **2020**, Doi: 10.1089/mdr.2020.0225.

[5] K. Zhu, S. Chen, T. Sysoeva, L. You, *PLoS Biol.* **2019**, *17*, e3000573.

[6] E. T. Arung, K. Shimizu, R. Kondo, *Planta Med.* **2006**, *72*, 847.

[7] E. T. Arung, K. Yoshikawa, K. Shimizu, R. Kondo, *Fitoterapia* **2010**, *81*, 120.

[8] T. Zhao, G. Yan, S. Pan, H. Wang, A. Hou, *Chem. Biodivers.* **2009**, *6*, 2209.

[9] L. Wang, Y. Yang, C. Liu, R. Chen, *J. Asian Nat. Prod. Res.* **2010**, *12*, 431.

[10] K. Likhitwitayawuid, B. Sritularak, W. De-Eknamkul, *Planta Med.* **2000**, *66*, 275.

[11] J. Sun, X. He, M. Zhao, L Li, C. Li, Y. Dong, *Molecules* **2014**, *19*, 13147.

[12] Q. Lin, J. Yuan, Z. Ma, J. Liang, X. Zhai, I. Khan, Y. Ding, G. Ren, *Nat. Prod. Commun.* **2016**, *11*, 1843.

[13] E.T. Arung, K. Shimizu, H. Tanaka, R. Kondo, *Lett. Drug Des. Discov.* **2010**, *7*, 602.

[14] F. Ferrari, I. Messana, M.C. Araujo, *Planta Med.* **1989**, *55*, 70.

[15] P.A. Segun, O.O. Ogbole, F.M.D. Ismail, L. Nahar, A.R. Evans, E.O.

Ajaiyeoba, S.D. Sarker, *Fitoterapia* **2019**, *134*, 151.
[16] M. Tanjung, E.H. Hakim, Elfahmi, J. Latip, *Nat. Prod. Commun.* **2012**, *7*, 1309.
[17] S. Fang, B. Shieh, R. Wu, C. Lin, *Phytochemistry* **1995**, *38*, 535.
[18] R. Miyata, M. Sahlan, Y. Ishikawa, H. Hashimoto, S. Honda, S. Kumazawa, *J. Nat. Prod.* **2019**, *82*, 205.
[19] Z. Zheng, K. Cheng, J. Chao, J. Wu, M. Wang, *Food Chem.* **2008**, *106*, 529.
[20] R. Miyata, M. Sahlan, Y. Ishikawa, H. Hashimoto, S. Honda, S. Kumazawa, *Nat. Prod. Res.* **2019**, *82*, 205.
[21] J. Du, C. Wang, J. Yang, X. He, X. Han, C. Li, X. Chai, Y. Wang, Y. Zhu, Z. Li, *Nat. Prod. Res.* **2019**, *33*, 673.
[22] Y. Cui, S. Taniguchi, T. Kuroda, T. Hatano, *Molecules* **2015**, *20*, 12500.
[23] Y. Liu, Q. Sun, L. Li, S. Song, *Biochem. Syst. Ecol.* **2019**, *87*, 103958.
[24] D.T.M. Huonga, L.T.N. Vu, L.T. Anh, N.T. Cuc, N.X. Nhiem, B.H. Tai, P.V. Kiem, M. Litaudon, T.D. Thach, C.V. Minh, V.C. Pham, *Phytochem. Lett.* **2019**, *34*, 39.
[25] K. Chen, Z. Hu, W. Song, Z. Wang, J. He, X. Shi, Q. Cui, X. Qiao, M. Ye, *ACS Synth. Biol.* **2019**, *8*, 1858.
[26] Z. Wang, S. Wang, Z. Xu, M. Li, K. Chen, Y. Zhang, Z. Hu, M. Zhang, Z. Zhang, X. Qiao, M. Ye, *Org. Lett.* **2019**, *21*, 2241.
[27] M. Kaouadji, S. Chiron, J. Garcia, F. Thomasson, M. Tissut, P. Ravanel, *Pergamon* **1992**, *31*, 2131.
[28] D.S. Jang, M. Cuendet, M.E. Hawthorne, L.B. Kardono, K. Kawanishi, H.H. Fong, R.G. Mehta, J.M. Pezzuto, A.D. Kinghorn, *Phytochemistry* **2002**, *61*, 867.
[29] M. Wang, W. Yang, X. Liu, Q. Liu, H. Zheng, X. Wang, T. Shen, S. Wang, D. Ren, *Nat. Prod. Res.* **2020**, *1*.
[30] J.Y. Kim, W.S. Lee, Y.S. Kim, M.J. Curtis-Long, B.W. Lee, Y.B. Ryu, K.H. Park, *J. Agr. Food Chem.* **2011**, *59*, 4589.
[31] Z. Zheng, K. Cheng, Q. Zhu, X. Wang, Z. Lin, M. Wang, *J. Agr. Food Chem.* **2010**, *58*, 5368.

[32] B.S. Simpson, D.J. Claudie, N.M. Smith, J.P. Gerber, R.A. Mckinnon, S.J. Semple, *Phytochemistry* **2011**, *72*, 1883.

[33] L. Zhang, J. Ji, C. Lei, H. Wang, Q. Zhao, A. Hou, *J. Nat. Prod.* **2012**, *75*, 699.

[34] Q. Jin, C. Lee, J.W. Lee, E.T. Yeon, D. Lee, S.B. Han, J.T. Hong, Y. Kim, M.K. Lee, B.Y. Hwang, *J. Nat. Prod.* **2014**, *77*, 1724.

[35] N.T. Dat, P.T.X. Binh, L.T.P. Quynh, C. Van Minh, H.T. Huong, J.J. Lee, *Fitoterapia* **2010**, *81*, 1224.

[36] S.M. Lee, Y.H. Song, Z. Uddin, Y.J. Ban, K.H. Park, *Rec. Nat. Prod.* **2017**, *11*, 514.

[37] J. Qin, M. Fan, J. He, X. Wu, L. Peng, J. Su, X. Cheng, Y. Li, L. Kong, R. Li, Q. Zhao, *Nat. Prod. Res.* **2015**, *29*, 1711.

[38] J.K. Cho, Y.B. Ryu, M.J. Curtis-Long, J.Y. Kim, D. Kim, S. Lee, W.S. Lee, K.H. Park, *Bioorg. Med. Chem. Lett.* **2011**, *21*, 2945.

[39] F. Ferrari, F. Delle Monache, A.I. Suarez, R.S. Compagnone, *Fitoterapia* **2000**, 71, 213.

[40] Z. Zheng, H. Tan, M. Wang, *Fitoterapia* **2012**, *83*, 1008.

[41] D. Wu, X. Zhang, X. Huang, X. He, G. Wang, W. Ye, *China J. Chin. Mater. Med.* **2010**, *35*, 1978.

[42] S. Li, W. Li, Y. Wang, Y. Asada, K. Koike, *Bioorg. Med. Chem. Lett.* **2010**, 20, 5398.

[43] Y. Lin, Y. Kuang, K. Li, S. Wang, W. Song, X. Qiao, G. Sabir, M. Ye, *Bioorgan. Med. Chem.* **2017**, *25*, 3706.

[44] N. Li, P. Zhang, H. Wu, J. Wang, F. Liu, W. Wang, *J. Funct. Foods* **2015**, *19*, 563.

[45] K. Kajiyana, S. Demizu, Y. Hiraga, K. Kinoshita, K. Koyama, K. Takahashi, Y. Tamura, K. Okada, T. Kinoshita, *J Nat. Prod.* **1992**, *55*, 1197.

[46] L.S.A. de Carvalho, R.B. Geraldo, J. de Moraes, P.L.S. Pinto, P. de F. Pinto, O. dos. S. Pereira Junior, A.A. da Silva Filho, *Exp. Parasitol.* **2015**, *159*, 207.

[47] H. Edziri, M. Mastouri, M.A. Mahjoub, Z. Mighri, A. Mahjoub, L. Verschaeve, *Molecules* **2012**, *17*, 7284.

[48] M. Li, X. Wu, X. Wang, T. Shen, D. Ren, *Nat. Prod. Res.* **2018**, *32,* 36.

[49] B. Pinto, A. Bertoli, C. Noccioli, S. Garritano, D. Reali, L. Pistelli, *Phytother. Res.* **2008**, *22*, 362.

[50] H.J. Lee, D.H. Lyu, U. Koo, K. Nam, S.S. Hong, K.O. Kim, K.H. Kim, D. Lee, W. Mar, *Arch. Pharm. Res.* **2012**, *35*, 163.

[51] L. Pistelli, A. Bertoli, I. Giachi, A. Manunta, *J. Nat. Prod.* **1998**, *61*, 1404.

[52] O.O. de Sayuri, L.A. de Souza, D.C. Baldoqui, M.H. Sarragiotto, A.A. Silva, *Química Nova* **2013**, *36*, 800.

[53] T. Fukai, B. Cai, K. Maruno, Y. Miyakawa, M. Konishi, T. Nomura, *Phytochemistry* **1998**, *49*, 2005.

[54] X. Hou, G. Ge, Z. Weng, Z. Dai, Y. Leng, L. Ding, L. Jin, Y. Yu, Y. Cao, J. Hou, *Bioorg. Chem.* **2018**, *80*, 577.

[55] E.T. Arung, K. Shimizu, R. Kondo, *Nat. Prod. Commun.* **2011**, *6*, 1397.

[56] J. He, P. Zhao, Z. Hu, S. Liu, Y. Kuang, M. Zhang, B. Li, C. Yun, X. Qiao, M. Ye, *Angew. Chem. Int. Ed. Engl* **2019**, *58*, 11513.

[57] S. Wu, F. Han, M. Song, S. Chen, Q. Li, Q. Zhang, K. Zhu, J. Shen, *J. Agr. Food Chem.* **2019**, *67*, 10222.

[58] X. Hou, G. Ge, Z. Weng, Z. Dai, Y. Leng, L. Ding, L. Jin, Y. Yu, Y. Cao, J. Hou, *Bioorg. Chem.* **2018**, *80*, 577.

[59] S. Wu, F. Han, M. Song, S. Chen, Q. Li, Q. Zhang, K. Zhu, J. Shen, *J. Agr. Food Chem.* **2019**, *67*, 10222.

[60] S. Wu, F. Han, M. Song, S. Chen, Q. Li, Q. Zhang, K. Zhu, J. Shen, *J. Agr. Food Chem.* **2019**, *67*, 10222.

[61] L. Yao, Y. Su, Z. Yin, N. Qin, T. Li, C. Si, E. Liu, X. Gao, *Holzforschung* **2010**, *64*, 571.

[62] M. Nagasaka, R. Hashimoto, Y. Inoue, K.I. Ishiuchi, M. Matsuno, Y. Itoh, M. Tokugawa, N. Ohoka, D. Morishita, H. Mizukami, T. Makino, H. Hayashi, *Molecules* **2018**, *23*, 1394.

[63] M. Bai, C. Zheng, L. Wu, S. Wu, Y. Cai, G. Chen, C. Han, X. Song, *Chem. Nat. Compd.* **2018**, *54*, 350.

[64] S. Mouffok, H.H.H.Y. Haba, C. Lavaud, C. Long, M. Benkhaled, *Rec. Nat.*

Prod. **2012**, *6*, 292.

[65] S.A. Ahmed, E.M. Kamel, *The Scientific World Journal* **2014**, *2014*, 1.

[66] P. Tsai, W. Huang, M. Hsieh, P. Sung, Y. Kuo, W. Wu, *Molecules* **2016**, *21*, 15.

[67] A.M. Karimov, T.N. Slobodyanyuk, E.K. Botirov, *Chem. Nat. Compd.* **2017**, *53*, 745.

[68] J. Li, Y. Wang, T.J. Smillie, I.A. Khan, *J. Pharmaceut. Biomed.* **2012**, *63*, 120.

[69] Y.N. Elkin, N.I. Kulesh, A.Y. Stepanova, A.I. Solovieva, V.M. Kargin, A.Y. Manyakhin, *J. Plant Physiol.* **2018**, *231*, 277.

[70] Y. Gong, X. Meng, Y. Zhu, *Biochem. Syst. Ecol.* **2018**, *80*, 122.

[71] Y. Fan, S. Yue, Z. Guo, L. Xin, C. Wang, D. Zhao, H. Guan, C. Wang, *Molecules* **2018**, *23*, 1361.

[72] Q. Wu, X. He, C. Jiang, W. Zhang, Z. Shi, H. Li, Y. Zhu, *Fitoterapia* **2018**, *124*, 113.

[73] J.P. Dzoyem, H. Hamamoto, B. Ngameni, B.T. Ngadjui, k. Sekimizu, *Drug Discov. Ther.* **2013**, *7*, 66.

[74] Q. Han, C. Qiao, J. Song, N. Yang, X. Cao, Y. Peng, D. Yang, S. Chen, H. Xu, *Chem. Biodivers.* **2007**, *4*, 940.

[75] V. Kuete, B. Ngameni, B. Wiench, B. Krusche, C. Horwedel, B.T. Ngadjui, T. Efferth, *Planta Med.* **2011**, *77*, 1984.

[76] J.P. Dzoyem, A.H.L. Nkuete, B. Ngameni, J.N. Eloff, *Arch. Pharm. Res.* **2017**, *40*, 1129.

[77] M.M. Radwan, M.A. Elsohly, D. Slade, S.A. Ahmed, L. Wilson, A.T. El-Alfy, I.A. Khan, S.A. Ross, *Phytochemistry* **2008**, *69*, 2627.

[78] S. Tahara, Y. Katagiri, J.L. Ingham, J. Mizutani, *Phytochemistry* **1994**, *36*, 1261.

[79] N.O.A. Omisore, C.O. Adewunmi, E.O. Iwalewa, B.T. Ngadjui, T.K. Adenowo, B.M. Abegaz, J.A. Ojewole, J. Watchueng, *Braz. J. Med. Biol. Res.* **2005**, *38*, 1087.

[80] W. Zhu, J. Qiu, Y. Zeng, P. Yi, H. Lou, J. Jian, M. Zuo, L. Duan, W. Gu, L.

Huang, Y. Li, C. Yuan, X. Hao, *Phytochemistry* **2019**, *164*, 33.
[81] W. Chiou, C. Lee, J. Liao, C. Chen, *Life Sci.* **2011**, *88*, 335.
[82] W. Chiou, C. Chen, B. Wei, *Ecid.-based Compl. Alt.* **2011**, *2011*, 1.
[83] K. Feng, R. Chen, K. Xie, D. Chen, B. Guo, X. Liu, J. Liu, M. Zhang, J. Dai, *Org. Biomol. Chem.* **2018**, *16*, 452.
[84] J.H. Kim, Y.B. Ryu, N.S. Kang, B.W. Lee, J.S. Heo, I.Y. Jeong, K.H. Park, *Biol. Pharm. Bull.* **2006**, *29*, 302.
[85] J.H. Kim, I.S. Cho, Y.K. So, H. Kim, Y.H. Kim, *J. Enzym. Inhib. Med. Ch.* **2018**, *33*, 1048.
[86] L. Ma, F. Xu, F. Li, J. Wang, M. Shang, G. Liu, S. Cai, *Biomed. Chromatogr.* **2016**, *30*, 175.
[87] D. Lee, K.P.L. Bhat, H.H.S. Fong, N.R. Farnsworth, J.M. Pezzuto, A.D. Kinghorn, *J. Nat. Prod.* **2001**, *64*, 1286.
[88] T. Fukai, M. Yonekawa, A. Hou, T. Nomura, H. Sun, J. Uno, *J. Nat. Prod.* **2003**, *66*, 1118.
[89] K. Akter, E.C. Barnes, W.L. Loa-Kum-Cheung, P. Yin, M. Kichu, J.J. Brophy, R.A. Barrow, I. Imchen, S.R. Vemulpad, J.F. Jamie, *J. Ethnopharmacol.* **2016**, *185*, 171.
[90] P. Erasto, G. Bojase-Moleta, R.R.T. Majinda, *Phytochemistry* **2004**, *65*, 875.
[91] S. Wei, L. Lu, Z. Ji, J. Zhang, W. Wu, *Chem. Nat. Compd.* **2012**, *48*, 484.
[92] A.E. Nkengfack, A.G. Azebaze, A.K. Waffo, Z.T. Fomum, M. Meyer, F.R. van Heerden, *Phytochemistry* **2001**, *58*, 1113.
[93] Y. Wang, A. Hou, G. Zhu, D. Chen, H. Sun, *Planta Med.* **2005**, *71*, 273.
[94] P. Innok, T. Rukachaisirikul, A. Suksamrarn, *Chem. Pharm. Bull.* **2009**, *57*, 993.
[95] C. Chang, C. Lin, S. Kadota, M. Hattori, T. Namba, *Phytochemistry* **1995**, *40*, 945.
[96] J. Cho, H. Park, G. Huh, S.S. Lee, M. Bang, K. Choi, C. Oh, S. Ko, S. Cho, K. Chai, J. Kim, N. Baek, *Food Sci. Biotechnol.* **2014**, *23*, 1815.
[97] X Li, N. Wang, M. S. Wong, S. C. C. Albert, X. Yao, *Chem. Pharm. Bull.* **2006**, *54*, 570.

[98] Z. Cao, Q. Zeng, H. Pei, L. Ren, H. Bai, R. Na, *Oncol. Lett.* **2016**, *11*, 3719.

[99] H. Sato, S. Tahara, J.L. Ingham, S.Z. Dziedzic, *Phytochemistry* **1995**, *39*, 673.

[100] S. Djiogue, M. Halabalaki, X. Alexi, D. Njamen, Z.T. Fomum, M.N. Alexis, A. Skaltsounis, *J. Nat. Prod.* **2009**, *72*, 1603.

[101] A. Wojakowska, K. Kułak, M. Jasiński, P. Kachlicki, S. Stawiński, M. Stobiecki, *Acta Physiol. Plant.* **2015**, *37*, 152.

[102] D.M. Biondi, C. Rocco, G. Ruberto, *J. Nat. Prod.* **2003**, *66*, 477.

[103] S. El-Masry, M.E. Amer, M.S. Abdel-Kader, H.H. Zaatout, *Phytochemistry* **2002**, *60*, 783.

[104] A.H. Tuan, D.T. Tuan, D.T. Trang, B.H. Tai, N.X. Nhiem, P.H. Yen, P.V. Kiem, C.V. Minh, Duc TM, H.K. Kang, Y.C. Kim, Y.H. Kim, *J. Asian Nat. Prod. Res.* **2017**, *19*, 510.

[105] Eerdunbayaer, M.A.A. Orabi, H. Aoyama, T. Kuroda, T. Hatano, *Molecules* **2014**, *19*, 13027.

[106] S. Kumar, A.S. Pathania, A.K. Saxena, R.A. Vishwakarma, A. Ali, S. Bhushan, *Chem. Biol. Interact.* **2013**, *205*, 128.

[107] M. Stobiecki, A. Staszków, A. Piasecka, P.M. Garcia-Lopez, F. Zamora-Natera, P. Kachlicki, *J. Nat. Prod.* **2010**, *73*, 1254.

[108] S. Toda, Y. Shirataki, *Pharm. Biol.* **2002**, *40*, 422.

[109] K. Akiyama, F. Tanigawa, T. Kashihara, H. Hayashi, *Phytochemistry* **2010**, *71*, 1865.

[110] K. Pranorm, R. Nijsiri, S.E. Ö, *Heterocycles* **2004**, *63*, 879.

[111] P. Khaomek, C. Ichino, A. Ishiyama, H. Sekiguchi, M. Namatame, N. Ruangrungsi, E. Saifah, H. Kiyohara, K. Otoguro, S. Omura, H. Yamada, *J. Nat. Med.* **2008**, *62*, 217.

[112] A.E. Nkengfack, A.K. Waffo, G.A. Azebaze, Z.T. Fomum, M. Meyer, B. Bodo, F.R. van Heerden, *J. Nat. Prod.* **2000**, *63*, 855.

[113] G. Xiao, G. Li, L. Chen, Z. Zhang, J. Yin, T. Wu, Z. Cheng, X. Wei, Z. Wang, *J. Chromatogr. A* **2010**, *34*, 5470.

[114] F. Cottiglia, L. Casu, L. Bonsignore, M. Casu, C. Floris, M. Leonti, J.

Gertsch, J. Heilmann, *Planta Med.* **2005**, *71*, 254.
[115] L. Pistelli, C. Noccioli, G. Appendino, F. Bianchi, O. Sterner, M. Ballero, *Phytochemistry* **2003**, *64*, 595.
[116] R.F. Souza, G.A. da Silva, A.C. Arruda, M.N. da Silva, A.S. Santos, D.P.A. Grisólia, M.B. Silva, C. Salgado, M.S.P. Arruda, *J. Brazil. Chem. Soc.* **2016**, *28*, 1132.
[117] M.S. Ali, M.I. Ali, P.A. Onocha, Z. Ahmed, *J. Asian Nat. Prod. Res.* **2011**, *13*, 182.
[118] D.M. Biondi, C. Rocco, G. Ruberto, *J. Nat. Prod.* **2005**, *68*, 1099.
[119] J. Ren, Q. Huang, Y. Xu, M. Yang, J. Yang, K. Hu, *Anti-Cancer Drug.* **2015**, *26*, 599.
[120] L. Pistelli, K. Spera, G. Flamini, S. Mele, I. Morelli, *Phytochemistry* **1996**, *42*, 1455.
[121] S. Wei, W. Wu, Z. Ji, *Int. J. Mol. Sci.* **2012**, *13*, 7375.
[122] G. Shen, D. Huhman, Z. Lei, J. Snyder, L.W. Sumner, R.A. Dixon, *Plant Physiol.* **2012**, *159*, 70.
[123] T. Fukai, A. Marumo, K. Kaitou, T. Kanda, S. Terada, T. Nomura, *Life Sci.* **2002**, *71*, 1449.
[124] H.N. Elsohly, A.S. Joshi, A.C. Nimrod, L.A. Walker, A.M. Clark, *Planta Med.* **2001**, *67*, 87.
[125] F. Sun, Q. Li, J. Xu, *Chem. Biodivers.* **2017**, *14*, 1600193.
[126] J.S. Jeon, S.M. Kim, H.J. Lee, B.H. Um, H.K. Kim, C.Y. Kim, *J. Liq. Chromatogr. R. T.* **2012**, *35*, 1607.
[127] S. Yin, C. Fan, Y. Wang, L. Dong, J. Yue, *Bioorgan. Med. Chem.* **2004**, *12*, 4387.
[128] A.E. Nkengfack, J. Kouam, T.W. Vouffo, M. Meyer, M.S. Tempesta, Z.T. Fomum, *Pergamon* **1994**, *35*, 521.
[129] A. Yenesew, B. Irungu, S. Derese, J.O. Midiwo, M. Heydenreich, M.G. Peter, *Phytochemistry* **2003**, *63*, 445.
[130] B.B. Lemes, L. Da Silva Miguêz, R.L. Santos, J.C. Da Rocha Bastos Serafim, J.M.R. Figueiredo, I.F. Tavares, M. de Godoi Pereira, E. de Jesus

Marquês, F. Da Silva Ramos, R.S. El-Bachá, A.I. Ribeiro, M.F. Das Graças Fernandes Da Silva, L.C. de Souza Neta, *Phytochem. Lett.* **2019**, *30*, 181.

[131] S.H. Shim, *J. Korean Soc. Appl. Biol. Chem.* **2009**, *52*, 568.

[132] S.R. Shah, C.I. Ukaegbu, H.A. Hamid, O.R. Alara, *Food Measure* **2018**, *12*, 1947.

[133] C. Na, S. Hong, Y. Choi, Y. Lee, S. Hong, J. Lim, B. Kang, S. Park, D. Lee, *Arch. Pharm. Res.* **2010**, *33*, 999.

[134] L. Luan, X. Shen, X. Liu, Y. Wu, M. Tan, *Biomed. Chromatogr.* **2018**, *32*, 4059.

[135] H. Liu, Y. Bai, Y. Chen, Y. Zhao, *China J. Chin. Mater. Med.* **2008**, *33*, 1410.

[136] S. Kaennakam, E.R. Sukandar, K. Rassamee, P. Siripong, S. Tip-Pyang, *Phytochem. Lett.* **2019**, *31*, 187.

[137] X. Wang, D. Wang, X. Wang, M. Khutsishvili, K. Tamanyan, G. Fayvush, D. Atha, Y. Zhang, R.P. Borris, *Planta Med.* **2019**, *85*, 225.

[138] C.A. Sabphon, P.B. Temkitthawon, K.C. Ingkaninan, P.C. Sawasdee, *Nat. Prod. Commun.* **2015**, *10*, 301.

[139] Y. Lu, S. Zhu, Y. He, C. Peng, Z. Wang, Q. Tang, *Int. J. Mol. Sci.* **2019**, *20*, 3396.

[140] J. Zhang, W. Zhu, J. Xu, W. Kitdamrongtham, A. Manosroi, J. Manosroi, H. Tokuda, M. Abe, T. Akihisa, F. Feng, *J. Ethnopharmacol.* **2018**, *214*, 37.

[141] S. Hong, J. Kwon, N.T. Hiep, S.J. Sim, N. Kim, K.H. Kim, D. Lee, W. Mar, *J. Funct. Foods* **2018**, *40*, 785.

[142] M. Babaoglu, M.R. Davey, J.B. Power, F. Sporer, M. Wink, *Plant cell, Tissue Organ Cult.* **2004**, *78*, 29.

[143] K.H. Yoon, K.J. Park, J. Yin, K.H. Yoon, J.Y. Lee, Y.J. Hwang, D.I. Lee, Y.W. Choi, M.W. Lee, *Rec. Nat. Prod.* **2016**, *10*, 441.

[144] A. Yenesew, J.O. Midiwo, M. Heydenreich, D. Schanzenbach, M.G. Peter, *Phytochemistry* **2000**, *55*, 457.

[145] E. Talla, E. Yankep, J. Tanyi Mbafor, *B. Chem. Soc. Ethiopia* **2014**, *28*, 155.

[146] J. Wandji, Z.T. Fomum, F. Tillequin, G. Baudouin, M. Koch, *Pergamon*

1994, *35*, 1573.

[147] E. Bae, M. Na, D. Njamen, J. Mbafor, Z. Fomum, L. Cui, D. Choung, B. Kim, W. Oh, J. Ahn, *Planta Med.* **2006**, *72*, 945.

[148] Y. Orazbekov, M.A. Ibrahim, S. Mombekov, R. Srivedavyasasri, U. Datkhayev, B. Makhatov, N.D. Chaurasiya, B.L. Tekwani, S.A. Ross, *Evid-Based Compl. Alt.* **2018**, *2018*, 1370368.

[149] X. Jiang, C. Cao, W. Sun, Z. Chen, X. Li, L. Nahar, S.D. Sarker, M.I. Georgiev, W. Bai, *Food Chem. Toxicol.* **2019**, *126*, 56.

[150] J. Kouam, X.S. Noundou, L.B. Kouitcheu Mabeku, A.M. Lannang, M.I. Choudhary, Z.T. Fomum, *B. Chem. Soc. Ethiopia* **2007**, *21*, 373.

[151] E. Talla, D. Njamen, J.T. Mbafor, Z.T. Fomum, A. Kamanyi, J. Mbanya, R.M. Giner, M.C. Recio, S. Máñez, J.L. Ríos, *J. Nat. Prod.* **2003**, *66*, 891.

[152] A. Tedasen, S. Sukrong, B. Sritularak, T. Srisawat, P. Graidist, *Biomed. Pharmacother.* **2016**, *81*, 235.

[153] P. Posri, J. Suthiwong, P. Takomthong, C. Wongsa, C. Chuenban, C. Boonyarat, C. Yenjai, *Nat. Prod. Res.* **2019**, *33*, 1115.

[154] P. Maximo, A. Lourenco, S.S. Feio, J.C. Roseiro, *Z Naturforsch C J Biosci* **2002**, *57*, 609.

[155] S. Sangmalee, A. Laorpaksa, B. Sritularak, S. Sukrong, *Biol. Pharm. Bull.* **2016**, *39*, 631.

[156] H. Yamamoto, P. Zhao, K. Inoue, *Phytochemistry* **2002**, *60*, 263.

[157] S. Deachathai, W. Mahabusarakam, S. Phongpaichit, W.C. Taylor, *Phytochemistry* **2005**, *66*, 2368.

[158] N. Chaichamnong, P. Temkitthawon, N. Khorana, P. Pitpakdeeanan, P. Taepavarapruk, N. Nuengchamnong, Y. Siriwattanasathien, A. Suksamrarn, K. Ingkaninan, *Planta Med.* **2018**, *84*, 1134.

[159] Y. H. Jo, K. M. Choi, Q. Liu, S. B. Kim, H. J. Ji, M. Kim, S. S. Shin, S. G. Do, E. Shin, G. Jung, H. S. Yoo, B. Y. Hwang, M. K. Lee, *Nutrients* **2015**, *7*, 10480.

[160] D.C. Bilanda, R.À.G. Bidingha, P.D. Djomeni Dzeufiet, Y.B. Fouda, R.F. Ngapout, Y. Tcheutchoua, P.E. Owona, S.C. Njonte Wouamba, L. Tanfack

Tatchou, T. Dimo, P. Kamtchouing, *J. Ethnopharmacol.* **2020**, *246*, 112200.
[161] A.J.Y. Gbaweng, H. Daïrou, S. Zingué, T. Emmanuel, A.T. Tchinda, M. Frédérich, J.T. Mbafor, *Nat. Prod. Res.* **2020**, *34*, 659.
[162] S. Nagumo, A. Fukuju, M. Takayama, M. Nagai, R. Yanoshita, Y. Samejima, *Biol. Pharm. Bull.* **1999**, *10*, 1144.
[163] S. Ahn, S. Park, Y.J. Lee, E. Cho, Y.K. Lim, X.M. Li, M. Choi, Y. Seo, J. Kook, *Caries Res.* **2015**, *49*, 78.
[164] G.R. Shin, S. Lee, S. Lee, S. Do, E. Shin, C.H. Lee, *Ind. Crop. Prod.* **2015**, *70*, 322.
[165] Y. Zhang, X. Li, X. Yao, M. Wong, *Arch. Pharm. Res.* **2008**, *31*, 1534.
[166] J. Lee, W. K. Oh, J. S. Ahn, Y. H. Kim, J. T. Mbafor, J. Wandji, Z. T. Fomum, *Planta Med.* **2009**, *75*, 268.
[167] J. R. Villinski, C. Bergeron, J. C. Cannistra, J. B. Gloer, C. M. Coleman, D. Ferreira, J. Azelmat, D. Grenier, S. Gafner, *J. Nat. Prod.* **2014**, *77*, 521.
[168] P. Gyoergy, K. Murata, H. Ikehata, *Nature* **1964**, *203*, 872.
[169] L. Guo, X. Yang, X. Sun, X. Wu, *Phytochem. Lett.* **2017**, *20*, 32.
[170] A. Hirota, M. Inaba, Y.C. Chen, N. Abe, S. Taki, M. Yano, S. Kawaii, *Biosci. Biotechnol. Biochem.* **2004**, *68*, 1372.
[171] Q. Luo, Q. Chen, Y. Wu, M. Jiang, Z. Chen, X. Zhang, H. Chen, *China J. Chin. Mater. Med.* **2010**, *35*, 2416.
[172] J. Bórquez, E.J. Kennelly, M.J. Simirgiotis, *Food Res. Int.* **2013**, *52*, 288.
[173] M. Iinuma, M. Ohyama, T. Tanaka, M. Mizuno, H. Soon-Keun, *Phytochemistry* **1993**, *33*, 1241.
[174] Z. Zhang, W. Yuan, P. Wang, G. Grant, S. Li, *Nat. Prod. Res.* **2011**, *25*, 1641.
[175] Y. Zhang, X. Xu, B. Hu, Q. Liu, C. Hou, Y. Liu, J. Yang, *Acta Pharm. Sin.* **1997**, *32*, 301.
[176] G. A. Lane, O. R. W. Sutherland, R. A. Skipp, *J. Chem. Ecol.* **1987**, *13*, 771.
[177] S. A. Adesany, M. J. O. A. Roberts, *Phytochemistry* **1985**, *24*, 2699.
[178] Y. Lin, Y. Kuang, K. Li, S. Wang, S. Ji, K. Chen, W. Song, X. Qiao, M. Ye, *Bioorgan. Med. Chem.* **2017**, *25*, 5522.

[179] M. Wang, W. Yang, X. Liu, Q. Liu, H. Zheng, X. Wang, T. Shen, S. Wang, D. Ren, *Nat. Prod. Res.* **2020**, *1*.

[180] V. Kuete, L.P. Sandjo, G.M.N. Kwamou, B. Wiench, A.E. Nkengfack, T. Efferth, *Phytomedicine* **2014**, *21*, 682.

[181] D. E. Djeussi, L. P. Sandjo, J. A. K. Noumedem, L. K. Omosa, B. T. Ngadjui, V. Kuete, *BMC Complem. Altern. M.* **2015**, *15*, 453.

[182] M. Chacha, G. Bojasemoleta, R. Majinda, *Phytochemistry* **2005**, *66*, 99.

[183] Q. Xu, Y. Hu, G. Li, W. Xu, Y. Zhang, X. Yang, *Molecules* **2018**, *23*, 614.

[184] H. Li, X. Meng, Y. Jiang, X. Lin, D. Xiong, D. Wang, H. Lee, L. Cui, *Phytochem. Lett.* **2018**, *28*, 130.

[185] C. Rodríguez-Pérez, R. Quirantes-Piné, N. Amessis-Ouchemoukh, K. Madani, A. Segura-Carretero, A. Fernández-Gutierrez, *J. Pharmaceut. Biomed.* **2013**, *77*, 167.

[186] T. Rukachaisirikul, P. Innok, N. Aroonrerk, W. Boonamnuaylap, S. Limrangsun, C. Boonyon, U. Woonjina, A. Suksamrarn, *J. Ethnopharmacol.* **2007**, *110*, 171.

[187] X. Yang, S. Deng, M. Huang, J. Wang, L. Chen, M. Xiong, J. Yang, S. Zheng, X. Ma, P. Zhao, Y. Feng, *Bioorg. Med. Chem. Lett.* **2017**, *27*, 1463.

[188] M. Mizuno, N. Matsuura, M. Iinuma, T. Tanaka, *Phytochemistry* **1992**, *31*, 643.

[189] M. Iinuma, M. Ohyama, T. Tanaka, *Phytochemistry* **1995**, *38*, 539.

[190] O. R. Alara, N. H. Abdurahman, C. I. Ukaegbu, N. H. Azhari, N. A. Kabbashi, *J. Liq. Chromatogr. R. T.* **2018**, *41*, 722.

[191] W. Lo, C. Wu, F. Chang, W. Wang, A. T. Khalil, K.H. Lee, Y. Wu, *Nat. Prod. Res.* **2003**, *17*, 91.

[192] M. Mizuno, T. Tanaka, N. Matsuura, M. Iinuma, C. Cheih, *Phytochemistry* **1990**, *29*, 2738.

[193] Y. Yang, Y. Zhang, Z. Chen, Y. Zhang, X. Yang, *Phytomedicine* **2018**, *38*, 166.

[194] C.F. Sampaio, N.R. Lucchetta, A.P.F. Punhagui, P.R. Banedetti, N.S. Arakawa, F.R.F. Seiva, G.S.A. Fernandes, *Microsc. Res. Techniq.* **2019**, *82*,

345.

[195] X. Liu, Y. Zhang, X. Yang, Y. Yang, W. Xu, W. Zhao, K. Peng, Y. Gong, N. Liu, P. Zhang, *Molecules* **2019**, *24,* 3750.

[196] R. Wen, H. Lv, Y. Jiang, P. Tu, *Planta Med.* **2018**, *84,* 1174.

[197] L. D. Dat, N. T. M. Tu, N. V. Duc, B. T. T. Luyen, C. T. T. Huyen, H. J. Jang, D. T. Thu, T. T. Huong, L. H. Tram, N. V. Thong, N. D. Hung, Y. H. Kim, N. P. Thao, *Carbohyd. Res.* **2019**, *484*, 107778.

[198] F. Kuang, Y. Liu, Y. Chen, R. Zhan, *Nat. Prod. Res.* **2020**, *34*, 390.

[199] S. Lam, Y. Li, P. Kuo, T. Hwang, M. Yang, C. Wang, J. Tzen, *Molecules* **2019**, *24*, 1371.

[200] Y. Hsu, W. Lai, C. Li, Y. Lan, Y. Tsai, A. Hunyadi, M. Hou, Y.S. Shiou, Y. Wu, F. Chang, *Nat. Prod. Commun.* **2016**, *11*, 1099.

[201] J. Yang, X. Zhou, X. Nong, J. Cao, Y. Hui, M. Wen, W. Chen, *Nat. Prod. Res.* **2020**, *9*, 1.

[202] Y. Cui, H. Yang, J. Jing, T. Liu, R. Wang, F. Di, F. Han, Y. Zhao, Z. Yu, *J. Pharmaceut. Biomed.* **2020**, *179*, 113029.

[203] M. Cao, H. Yuan, M. Daniyal, H. Yu, Q. Xie, Y. Liu, B. Li, Y. Jian, C. Peng, D. Tan, Y. Peng, M.I. Choudhary, A. Rahman, W. Wang, *Fitoterapia* **2019**, *138*, 104276.

[204] J. Kwon, N.T. Hiep, D.W. Kim, S. Hong, Y. Guo, B.Y. Hwang, H.J. Lee, W. Mar, D. Lee, *J. Nat. Prod.* **2016**, *79*, 1938.

[205] Y. Zhang, D. Zhou, W. Liu, C. Li, L. Hao, G. Zhang, S. Deng, R. Yang, J. Qin, J. Li, W. Deng, *Chem. Biodivers.* **2019**, *16*, 1800465.

[206] B. Zhou, C.X. Wan, *J. Asian Nat. Prod. Res.* **2015**, *17*, 256.

[207] D.W. Kim, H.S. Woo, J.Y. Kim, J.A. Ryuk, K.H. Park, B.S. Ko, *J. Enzyme Inhib. Med. Chem.* **2016**, *31*, 742.

[208] X. Yu, S.M. Li, *Chembiochem.* **2011**, *12*, 2280.

[209] Y. Fan, R. Ye, H. Xu, X. Feng, C. Ma, *J. Food Sci.* **2019**, *84*, 1224.

[210] K. W. Kim, T. H. Quang, W. Ko, D. C. Kim, C. S. Yoon, H. Oh, Y. C. Kim, *Pharm. Biol.* **2018**, *56,* 192.

[211] W. Quan, L. Hee, K. Chul, N. Chu, U. Byung, O. Min-Ho, K. Kyeong-Man,

Planta Med. **2008**, *74*, 168.

[212] W. Lo, F. Chang, T. Hsieh, Y. Wu, *Phytochemistry* **2002**, *60*, 839.

[213] M. Bobrowska-Hagerstrand, A. Wrobel, L. Mrowczynska, T. Soderstrom, Y. Shirataki, N. Motohashi, J. Molnar, K. Michalak, H. Hagerstrand, *Oncol. Res.* **2003**, *13*, 463.

[214] W. Kitdamrongtham, K. Ishii, K. Ebina, J. Zhang, M. Ukiya, K. Koike, H. Akazawa, A. Manosroi, J. Manosroi, T. Akihisa, *Chem. Biodivers.* **2014**, *11*, 73.

[215] B. A. Schütz, A. D. Wright, T. Rali, O. Sticher, *Phytochemistry* **1995**, *40*, 1273.

[216] T. Tanaka, M. linuma, F.Asai, M. Ohyama, C. L. Burandt, *Phytochemistry* **1997**, *46*, 1431.

[217] X. Wang, L. Zhen, G. Zhang, M. Wong, L. Qin, X. Yao, *Phytomedicine* **2011**, *18*, 868.

[218] Q. Ye, X. Ma, C. Hu, B. Lin, L. Xu, C. Zheng, L. Qin, *Phytomedicine* **2015**, *22*, 94.

[219] J. Du, Z. He, R. Jiang, W. Ye, H. Xu, P.P. But, *Phytochemistry* **2003**, *62*, 1235.

[220] H. Sasaki, Y. Kashiwada, H. Shibata, Y. Takaishi, *Phytochemistry* **2012**, *82*, 136.

[221] H. Sasaki, H. Shibata, K. Imabayashi, Y. Takaishi, Y. Kashiwada, *J. Agr. Food Chem.* **2014**, *62*, 6345.

[222] M. Iinuma, M. Ohyama, T. Tanaka, F.A. Lang, *J. Nat. Prod.* **1993**, *56*, 2212.

[223] F. Hiroshi, G. Katsumi, T. Mamoru, *Chem. Pharm. Bull.* **1988**, *36*, 4174.

[224] Y. Li, J. Ning, Y. Wang, C. Wang, C. Sun, X. Huo, Z. Yu, L. Feng, B. Zhang, X. Tian, X. Ma, *Toxicol. Lett.* **2018**, *294*, 27.

[225] J. W. Lee, J. H. Lee, C. Lee, Q. Jin, D. Lee, Y. Kim, J.T. Hong, M. K. Lee, B.Y. Hwang, *Bioorg. Med. Chem. Lett.* **2015**, *25*, 960.

[226] H. N. Elsohly, A. S. Joshi, A. C. Nimrod, L. A. Walker, A. M. Clark, *Planta Med.* **2001**, *67*, 87.

[227] M. Maya, Y. Hiromi, W. Tsutomu, M. Toshio, *J. Nat. Prod.* **2009**, *72*, 63.

[228] N. O. Omisore, C. O. Adewunmi, E. O. Iwalewa, B. T. Ngadjui, T. K. Adenowo, B. M. Abegaz, J. A. Ojewole, J. Watchueng, *Braz. J. Med. Biol. Res.* **2005**, *38*, 1087.

[229] M. Lin, D. Xiang, X. Chen, H. Huo, *J. Agr. Food Chem.* **2019**, *67*, 8291.

[230] J. Y. Park, J. A. Ko, D. W. Kim, Y. M. Kim, H. J. Kwon, H. J. Jeong, C. Y. Kim, K. H. Park, W. S. Lee, Y. B. Ryu, *J. Enzyme Inhib. Med. Chem.* **2016**, *31*, 23.

[231] K. Kawabata, Y. Kato, T. Sakano, N. Baba, K. Hagiwara, A. Tamura, S. Baba, M. Natsume, H. Ohigashi, *Biosci. Biotechnol. Biochem.* **2015**, *79*, 799.

[232] D. Nikolic, R.B. van Breemen, *Curr. Anal. Chem.* **2013**, *9*, 71.

[233] S. Venturelli, M. Burkard, M. Biendl, U. M. Lauer, J. Frank, C. Busch, *Nutrition* **2016**, *32*, 1171.

[234] I. L. Paraiso, L. S. Plagmann, L. Yang, R. Zielke, A. F. Gombart, C. S. Maier, A. E. Sikora, P. R. Blakemore, J. F. Stevens, *Mol. Nutr. Food Res.* **2019**, *63*, 1800923.

[235] T. Maliar, P. Nemeček, E. ürgeová, M. Maliarová, V. Nesvadba, K. Krofta, K. Vulganová, E. Krošlák, J. Kraic, *Chem. Pap.* **2017**, *71*, 41.

[236] B. Ngameni, M. Touaibia, R. Patnam, A. Belkaid, P. Sonna, B.T. Ngadjui, B. Annabi, R. Roy, *Phytochemistry* **2006**, *67*, 2573.

[237] T. Fukai, J. Nishizawa, T. Nomura, *Pergamon.* **1994**, *35*, 515.

[238] J. Chen, M. Cheng, C. Shu, P. Sung, Y. Lim, L. Cheng, S. Wang, L. Chen, *Chem. Nat. Compd.* **2017**, *53*, 632.

[239] X. Wang, *J. Intel. Mat. Syst. Str.* **2017**, *29*, 255.

[240] K. Kajiyama, S. Demizu, Y. Hiraga, K. Kinoshita, K. Koyama, K. Takahashi, Y. Tamura, K. Okada, T. Kinoshita, *Pergamon* **1992**, *31*, 3229.

[241] C. Simmler, D.C. Lankin, D. Nikolić, R.B. van Breemen, G.F. Pauli, *Fitoterapia* **2017**, *121*, 6.

[242] R. Muharini, A. Díaz, W. Ebrahim, A. Mándi, T. Kurtán, N. Rehberg, R. Kalscheuer, R. Hartmann, R.S. Orfali, W. Lin, Z. Liu, P. Proksch, *J. Nat. Prod.* **2016**, *80*, 169.

[243] J. Lee, H. Mei, I. Kuo, T. Lee, Y. Chen, C. Lee, *Molecules* **2019**, *24*, 3297.

[244] F. S. Wahyuni, D. A. I. Ali, N. H. Lajis, Dachriyanus, *Pharmacogn. J.* **2016**, *9*, 55.

[245] B. A. Tonali, L. H. Rafael, M. S. L. Elizabrth, R. C. Ricardo, R. L. Edgar, P. Benjamin, N. M. C. Omar, S. C. Laura, P. Enrique, C. Trejo, S. A. Daniela, P. C. Jose, R. Camilo, P. Veronica, T. r. Monica, *BMC Complement Altern. Med.* **2013**, *13*, 262.

[246] R. Achara, S. Tawanun, M. Wisanu, *Nat. Prod. Commun.* **2015**, *10*, 1969.

[247] M. Tanjung, T. S. Tjahjandarie, R. D. Saputri, B. D. Kurnia, M. F. Rachman, Y. M. Syah, *Nat. Prod. Res.* **2021**, *35*, 407.

[248] N. Kheyar-Kraouche, A. B. Da Silva, A. T. Serra, F. Bedjou, M. R. Bronze, *J. Pharmaceut. Biomed.* **2018**, *156*, 297.

[249] S. S. Teh, G. C. L. Ee, S. H. Mah, *Asian Journal of Chemistry* **2013**, *25*, 8780.

[250] C. Ito, T. Matsui, A. Niimi, H.T. Tan, M. Itoigawa, *Planta Med.* **2017**, *83*, 812.

[251] H. T. Vo, N. T. N. Ngo, T. Q. Bui, H. D. Pham, L. D. Nguyen, *Phytochem. Lett.* **2015**, *13*, 119.

[252] P. Nugitrangson, S. C. Puthong, T. Iempridee, W. Pimtong, S. Pornpakakul, C. Chanchao, *Exp. Biol. Med.* **2016**, *241*, 166.

[253] A. Aravind, R. Pandey, B. Kumar, K. Asha, K.B. Rameshkumar, *Nat. Prod. Commun.* **2016**, *11*, 1839.

[254] N. Jamila, M. Khairuddean, S.N. Khan, N. Khan, H. Osman, *Rec. Nat. Prod.* **2014**, *8*, 312.

[255] E. R. Sukandar, S. Kaennakam, K. Rassamee, T. Ersam, P. Siripong, S. Tip-Pyang, *J. Nat. Prod.* **2019**, *82*, 1312.

[256] M. Zhou, K. Zhou, Y. Zhao, N. Xiang, T. Zhang, Y. Wang, W. Dong, B. Ji, L. Li, J. Lou, G. Li, Q. Hu, *Phytochem. Lett.* **2015**, *11*, 245.

[257] B. T. D. Trinh, T. T. T. Quach, D. N. Bui, D. Staerk, L. D. Nguyen, A. K. Jäger, *Fitoterapia* **2017**, *118*, 126.

[258] N. K. Nguyen, X. A. Truong, T. Q. Bui, D. N. Bui, H. X. Nguyen, P. T. Tran, L. D. Nguyen, *Chem. Biodivers.* **2017**, *14*, 1700232.

[259] M. Y. Ibrahim, N. M. Hashim, S. Mohan, M. A. Abdulla, S. I. Abdelwahab, I. A. Arbab, M. Yahayu, L. Z. Ali, O. E. Ishag, *Arab. J. Chem.* **2015**, *8*, 129.

[260] M. S. Meah, M. Lertcanawanichakul, P. Pedpradab, W. Lin, K. Zhu, G. Li, P. Panichayupakaranant, *Lett. Appl. Microbiol.* **2020**, *71*, 510.

[261] Z. Na, Y. Xu, *Nat. Prod. Res.* **2010**, *24*, 1648.

[262] Z. Na, Q. Song, B. I. H. Hua, *Rec. Nat. Prod.* **2013**, *7*, 220.

[263] Y. Duan, Y. Dai, G. Wang, X. Zhang, H. Chen, J. Chen, X. Yao, X. Zhang, *J. Nat. Prod.* **2010**, *73*, 1283.

[264] H. Zhang, D. Zhang, Y. Lao, W. Fu, S. Liang, Q. Yuan, L. Yang, H. Xu, *J. Nat. Prod.* **2014**, *77*, 2148.

[265] O. Tusevski, J. Petreska Stanoeva, M. Stefova, D. Pavokovic, S. Gadzovska Simic, *Acta Physiol. Plant.* **2014**, *36*, 2555.

[266] G. A. Mohamed, A. M. Al-Abd, A. M. El-Halawany, H. M. Abdallah, S. R. M. Ibrahim, *J. Ethnopharmacol.* **2017**, *198*, 302.

[267] W. Schmidt, A.M. Abd el-Mawla, J.L. Wolfender, K. Hostettmann, L. Beerhues, *Planta Med.* **2000**, 66, 380.

[268] Q. Han, N. Yang, H. Tian, C. Qiao, J. Song, D.C. Chang, S. Chen, K.Q. Luo, H. Xu, *Phytochemistry* **2008**, *69*, 2187.

[269] Q. Han, C. Qiao, J. Song, N. Yang, X. Cao, Y. Peng, D. Yang, S. Chen, H. Xu, *Chem. Biodivers.* **2007**, *4*, 940.

[270] S. Niu, Z. Li, F. Ji, G. Liu, N. Zhao, X. Liu, Y. Jing, H. Hua, *Phytochemistry* **2012**, *77*, 280.

[271] F. Ji, Z. Lin, G. Liu, S. Niu, N Zhang, X. Liu, H. Hua, *Nat. Prod. Commun.* **2012**, *7*.

[272] P. Li, H. Anandhisenthilkumar, S. Wu, B. Liu, Z. Guo, J.E. Fata, E.J. Kennelly, C. Long, *J. Chromatogr. B* **2016**, *1011*, 179.

[273] Y. Li, P. Zhao, Y. Chen, Y. Fu, K. Shi, L. Liu, H. Liu, M. Xiong, Q. Liu, G. Yang, Y. Xiao, *Bioorgan. Med. Chem.* **2017**, *25*, 6605.

[274] Q. Hu, G. Li, M. Zhou, K. Zhou, Y. Zhao, N. Xiang, T. Zhang, Y. Wang, W. Dong, B. Ji, J. Lou, L. Li, C. Zhang, *Heterocycles* **2015**, *91*, 604.

[275] Z. Wang, Y. Lin, T. Li, F. Dai, G. Luo, G. Xiao, C. Tang, *Int. J. Food Prop.*

2019, *22*, 1340.

[276] N. Khamthong, N. Hutadilok-Towatana, *Nat. Prod. Commun.* **2017**, *12*, 453.

[277] A. Han, J. Kim, D. D. Lantvit, L. B. S. Kardono, S. Riswan, H. Chai, E. J. Carcache De Blanco, N. R. Farnsworth, S. M. Swanson, A. D. Kinghorn, *J. Nat. Prod.* **2009**, *72*, 2028.

[278] E. T. Ngouamegne, R. S. Fongang, S. Ngouela, F. F. Boyom, M. Rohmer, E. Tsamo, J. Gut, P.J. Rosenthal, *Chem. Pharm. Bull.* **2008**, *56*, 374.

[279] B. Ovalle-Magallanes, D. Eugenio-Pérez, J. Pedraza-Chaverri, *Food Chem. Toxicol.* **2017**, *109*, 102.

[280] S. Wang, Q. Li, M. Jing, E. Alba, X. Yang, R. Sabaté, Y. Han, R. Pi, W. Lan, X. Yang, J. Chen, *Neurochem. Res.* **2016**, *41*, 1806.

[281] L. D. Nguyen, H. T. Vo, H. D. Pham, J. D. Connolly, L. J. Harrison, *Phytochemistry* **2003**, *63*, 467.

[282] K. Trisuwan, T. Ritthiwigrom, *Arch. Pharm. Res.* **2012**, *35*, 1733.

[283] W. Jing, C Jiang, F. Ji, H. Hua, Z. Li, *J. Asian Nat. Prod. Res.* **2013**, *15*, 1152.

[284] S. R. M. Ibrahim, H. M. Abdallah, A. M. El-Halawany, M. F. Radwan, I. A. Shehata, E. M. Al-Harshany, M. F. Zayed, G. A. Mohamed, *Phytochem. Lett.* **2018**, *25*, 12.

[285] S. Ibrahim, H. M. Abdallah, A. M. El-Halawany, A. M. Nafady, G. A. Mohamed, *Nat. Prod. Res.* **2019**, *33*, 258.

[286] G. A. Mohamed, S. R. M. Ibrahim, M. I. A. Shaaban, S. A. Ross, *Fitoterapia* **2014**, *98*, 215.

[287] B. Vemu, M.C. Nauman, J.P. Veenstra, J.J. Johnson, *Int. J. Nutr.* **2019**, *4*, 38.

[288] H.S. Chae, E.Y. Kim, L. Han, N.R. Kim, B. Lam, J.H. Paik, K.D. Yoon, Y.H. Choi, Y.W. Chin, *Eur. J. Lipid Sci. Tech.* **2016**, *118*, 1416.

[289] H. Minami, E. Takahashi, M. Kodama, Y. Fukuyama, *Phytochemistry* **1996**, *41*, 629.

[290] S. Sanpa, M. Popova, V. Bankova, T. Tunkasiri, S. Eitssayeam, P. Chantawannakul, *PLoS One* **2015**, *10*, 126886.

[291] J. Ngoupayo, T.K. Tabopda, M.S. Ali, *Bioorgan. Med. Chem.* **2009**, *17*,

5688.

[292] B. Zhang, W. Fu, R. Wu, J. Yang, C. Yao, B. Yan, H. Tan, C. Zheng, Z. Song, H. Xu, *Planta Med.* **2019**, *85*, 444.

[293] S. F. Mamat, K. A. Azizan, S. N. Baharum, N. M. Noor, W. M. Aizat, *Sci. Hortic.* **2020**, *262*, 109004.

[294] A. Groweiss, J.H. Cardellina, M.R. Boyd, *J. Nat. Prod.* **2000**, *63*, 1537.

[295] L.N. Mariano, D.B. Vendramini-Costa, A.L. Ruiz, J.E. de Carvalho, R. Correa, F.V. Cechinel, M.F. Delle, R. Niero, *Pharm. Biol.* **2016**, *54*, 1697.

[296] C. Tantapakul, W. Phakhodee, T. Ritthiwigrom, S. Cheenpracha, U. Prawat, S. Deachathai, S. Laphookhieo, *J. Nat. Prod.* **2012**, *75*, 1660.

[297] T. Sriyatep, C. Tantapakul, R.J. Andersen, B.O. Patrick, S.G. Pyne, C. Muanprasat, S. Seemakhan, S. Borwornpinyo, S. Laphookhieo, *Fitoterapia* **2018**, *124*, 34.

[298] X. Liu, X. Hu, X. Peng, Y. Wang, X. Huang, Y. Zan, D. Li, Z. Li, H. Hua, *Bioorg. Chem.* **2020**, *94*, 103370.

[299] J. Nontakham, N. Charoenram, W. Upamai, M. Taweechotipatr, S. Suksamrarn, *Arch. Pharm. Res.* **2014**, *37*, 972.

[300] Y. Ji, R. Zhang, C. Zhang, X. Li, A. Negrin, C. Yuan, E.J. Kennelly, C. Long, *Molecules* **2019**, *24*,3568.

[301] G. Zuo, J. An, J. Han, Y. Zhang, G. Wang, X. Hao, Z. Bian, *Int. J. Mol. Sci.* **2012**, *13*, 8210.

[302] D.A. Mulholland, E.M. Mwangi, N.C. Dlova, N. Plant, N.R. Crouch, P.H. Coombes, *J. Ethnopharmacol.* **2013**, *149*, 570.

[303] G. Rath, O. Potterat, S. Mavi, K. Hostettmann, *Phytochemistry* **1996**, *43*, 513.

[304] L. Wang, Z. Li, H. Hua, X. Liu, *China J. Chin. Mater. Med.* **2008**, *33*, 2350.

[305] T.Q. Bui, A.T. Bui, K.T. Nguyen, V.T. Nguyen, B.T. D. Trinh, L.D. Nguyen, *Tetrahedron Lett.* **2016**, *57*, 2524.

[306] A. Lavaud, P. Richomme, M. Litaudon, R. Andriantsitohaina, D. Guilet, *J. Nat. Prod.* **2013**, *76*, 2246.

[307] V. Kuete, J.R. Nguemeving, V.P. Beng, A.G.B. Azebaze, F. Etoa, M. Meyer,

B. Bodo, A.E. Nkengfack, *J. Ethnopharmacol.* **2007**, *109*, 372.

[308] K.W. Wong, G.C.L. Ee, I.S. Ismail, T. Karunakaran, V.Y.M. Jong, *Nat. Prod. Res.* **2017**, *31*, 2513.

[309] J.R. Nguemeving, A.G. Azebaze, V. Kuete, C.N. Eric, V.P. Beng, M. Meyer, A. Blond, B. Bodo, A.E. Nkengfack, *Phytochemistry* **2006**, *67*, 1341.

[310] I. Ramírez-Gonzáilez, J.M. Amaro-Luis, A. Bahsas, *Nat. Prod. Commun.* **2013**, *8*, 1731.

[311] Q. Wu, S. Wang, L. Du, J. Yang, P. Xiao, *Phytochemistry* **1998**, *49*, 1395.

[312] Y.H. Jo, S.B. Kim, J.H. Ahn, A. Turk, E. Kwon, M. Kim, B.Y. Hwang, M.K. Lee, *Bioorg. Chem.* **2019**, *92*, 103234.
